# The Suf Iron-Sulfur Cluster Synthesis Pathway Is Required for Apicoplast Maintenance in Malaria Parasites

**DOI:** 10.1371/journal.ppat.1003655

**Published:** 2013-09-26

**Authors:** Jolyn E. Gisselberg, Teegan A. Dellibovi-Ragheb, Krista A. Matthews, Gundula Bosch, Sean T. Prigge

**Affiliations:** 1 Department of Biochemistry and Molecular Biology, Johns Hopkins Bloomberg School of Public Health, Baltimore, Maryland, United States of America; 2 W. Harry Feinstone Department of Molecular Microbiology and Immunology, Johns Hopkins Bloomberg School of Public Health, Baltimore, Maryland, United States of America; Robert-Koch-Institut, Berlin, Germany

## Abstract

The apicoplast organelle of the malaria parasite *Plasmodium falciparum* contains metabolic pathways critical for liver-stage and blood-stage development. During the blood stages, parasites lacking an apicoplast can grow in the presence of isopentenyl pyrophosphate (IPP), demonstrating that isoprenoids are the only metabolites produced in the apicoplast which are needed outside of the organelle. Two of the isoprenoid biosynthesis enzymes are predicted to rely on iron-sulfur (FeS) cluster cofactors, however, little is known about FeS cluster synthesis in the parasite or the roles that FeS cluster proteins play in parasite biology. We investigated two putative FeS cluster synthesis pathways (Isc and Suf) focusing on the initial step of sulfur acquisition. In other eukaryotes, these proteins can be located in multiple subcellular compartments, raising the possibility of cross-talk between the pathways or redundant functions. In *P. falciparum*, SufS and its partner SufE were found exclusively the apicoplast and SufS was shown to have cysteine desulfurase activity in a complementation assay. IscS and its effector Isd11 were solely mitochondrial, suggesting that the Isc pathway cannot contribute to apicoplast FeS cluster synthesis. The Suf pathway was disrupted with a dominant negative mutant resulting in parasites that were only viable when supplemented with IPP. These parasites lacked the apicoplast organelle and its organellar genome – a phenotype not observed when isoprenoid biosynthesis was specifically inhibited with fosmidomycin. Taken together, these results demonstrate that the Suf pathway is essential for parasite survival and has a fundamental role in maintaining the apicoplast organelle in addition to any role in isoprenoid biosynthesis.

## Author Summary

Iron is essential for the survival of blood stage *P. falciparum* and is used primarily in the synthesis of iron-sulfur (FeS) cluster cofactors. We investigated the role that (FeS) clusters play in malaria parasites. We demonstrated that the synthesis of FeS clusters is partitioned between two organelles: the Isc pathway is mitochondrial while the Suf pathway is found exclusively in the apicoplast organelle. Attempts to interfere with the Suf pathway through a dominant negative approach were only successful when parasite cultures were supplemented with an isoprenoid product. This result demonstrates that isoprenoid biosynthesis depends on a functional Suf pathway. Unexpectedly, we also observed the complete loss of the apicoplast organelle when we disrupted the Suf pathway. This phenotype does not result from inhibition of isoprenoid biosynthesis; we treated parasites with high levels of the isoprenoid inhibitor fosmidomycin without any loss of the apicoplast organelle. These results demonstrate that the Suf pathway has a fundamental role in maintaining the apicoplast organelle in addition to any role in isoprenoid biosynthesis. Inhibition of the Suf pathway, which is not found in humans, will block the growth of malaria parasites.

## Introduction

Iron-sulfur (FeS) clusters are ancient protein cofactors found in most organisms. These cofactors have a variety of roles including the transfer of single electrons, donation of sulfur atoms, initiation of free radical chemistry, oxygen sensing, and purely structural roles [1], [2]. FeS clusters are found in a variety of forms, but the most common are cubane 4Fe-4S, cuboidal 3Fe-4S, and binuclear 2Fe-2S clusters [3]. Proteins typically bind these clusters through cysteine residues, although other amino acids have been shown to be involved in coordinating the cofactor [1].

**Figure 1:**
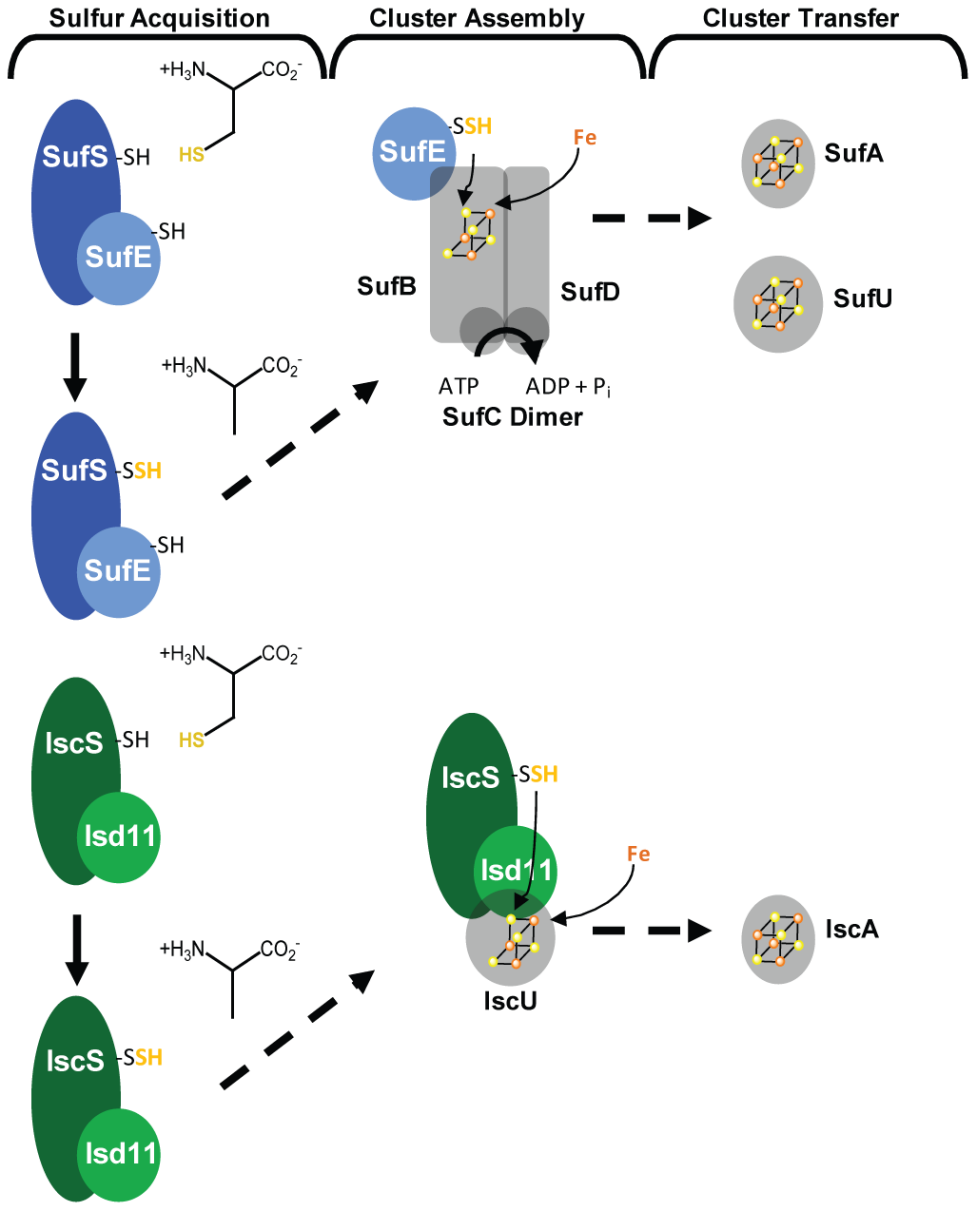
FeS cluster synthesis pathways. Both the Suf (above, blue) and Isc (below, green) pathways follow the same general steps of FeS cluster synthesis: sulfur mobilization, cluster assembly, and cluster transfer.

Proteins containing FeS clusters are typically sensitive to oxygen and the clusters rapidly degrade in extracellular environments. Thus, clusters are synthesized *de novo* by one of three known FeS biosynthetic pathways. The Nif pathway, the first synthesis pathway described, is primarily found in nitrogen-fixing bacteria [4]. The Isc and Suf pathways are the dominant FeS cluster synthesis pathways found in eukaryotes, and are also present in bacteria and archaea [5], [6]. In eukaryotes, the Isc pathway is mitochondrial [5] while the Suf pathway has thus far been found in species harboring a plastid organelle and has been localized to the chloroplast in *Arabidopsis thaliana* [7], [8]. The protozoan parasite *Blastocystis*, which lacks a plastid, contains components of the Suf pathway in the cytosol [9]. While the protein components of the Isc and Suf machinery are quite different, both pathways follow the same basic steps of sulfur mobilization, cluster assembly, and cluster transfer (**Figure 1**).

The Isc and Suf systems both depend on a cysteine desulfurase to mobilize sulfur from L-cysteine. The cysteine desulfurases of the eukaryotic Isc pathway (IscS) and of the Suf pathway (SufS) are only active when in complex with a partner protein (**Figure 1**). Isd11, a component of eukaryotic Isc pathways, is essential for mitochondrial FeS cluster synthesis in *Saccharomyces cerevisiae* and *Trypanosoma brucei* [10], [11], [12] but is not present in prokaryotes [13]. In the absence of Isd11, yeast IscS is prone to aggregation [10], [11]. Isd11 has a conserved LYK/R motif that is essential for its ability to activate IscS cysteine desulfurase activity [14]. SufS is activated by SufE, an accessory protein which is found in both prokaryotic and eukaryotic Suf pathways. Unlike Isd11, SufE forms a persulfide bond with the mobilized sulfur atom and acts to transfer the persulfide sulfur to the SufBCD assembly machinery [15]. Bacterial SufE has been shown to accelerate the cysteine desulfurase activity of SufS [16], [17]. In the presence of SufE, the V_max_ of *Escherichia coli* SufS is increased eight fold and an additional rate enhancement of 32 fold is observed when the assembly machinery (SufBCD complex) is present to accept the sulfur from SufE [16]. In *E. coli*, SufE does not interact with the Isc cysteine desulfurase [16] while in *A. thaliana* SufE has been shown to localize to both mitochondria as well as chloroplasts and serves to activate both cysteine desulfurases [18].

Malaria parasites harbor a plastid organelle called the apicoplast that is thought to have arisen from two sequential endosymbiotic events [19]. The apicoplast harbors biochemical pathways of prokaryotic origin such as type II fatty acid synthesis (FASII), lipoate synthesis, tRNA modification, and 2-*C*-methyl-D-erythritol 4-phosphate (MEP) isoprenoid biosynthesis [20]. Enzymes in these pathways are predicted to require FeS cluster cofactors. In prokaryotes, lipoate synthase (LipA), the tRNA modification enzyme MiaB, as well as the MEP enzymes IspG and IspH contain 4Fe-4S clusters [21], [22], [23], [24], [25]. The activity of these FeS proteins is in turn thought to be dependent on the 2Fe-2S electron transfer protein ferredoxin (Fd) [26], [27]. In malaria parasites, only Fd and IspH have thus far been shown to contain FeS clusters [27].

The MEP isoprenoid biosynthesis pathway, the target of the antimalarial fosmidomycin, was recently shown to be essential for the survival of erythrocytic stage malaria parasites [28], [29]. Parasites cultured in the presence of the MEP pathway product IPP (isopentenyl pyrophosphate) were no longer sensitive to fosmidomycin. Additionally, supplementation with IPP allowed parasites to survive without the apicoplast organelle, demonstrating that isoprenoids are the only metabolites produced in the apicoplast that are needed outside the organelle [28]. FeS cluster proteins are likely required for the production of essential isoprenoids. However, the synthesis of FeS clusters themselves has not been well characterized in malaria parasites. Only the *P. falciparum* SufC protein, part of the SufBCD assembly complex, has been studied to date, and was demonstrated to be an active ATPase localized to the apicoplast [30].

In this report, we investigated two putative FeS cluster synthesis pathways (Isc and Suf), focusing on the initial step of sulfur acquisition. In *P. falciparum*, SufS and its partner SufE were found exclusively in the apicoplast and SufS was shown to have cysteine desulfurase activity in a complementation assay. IscS and its effector Isd11 were solely mitochondrial, suggesting that the Isc pathway does not contribute to apicoplast FeS cluster synthesis. We disrupted the Suf pathway using a dominant negative mutant of SufC and showed that these parasites only survive when cultured in the presence of IPP. Furthermore, these parasites lack the apicoplast organelle and its organellar genome – a phenotype not observed when isoprenoid biosynthesis was specifically inhibited with fosmidomycin. Taken together, these results demonstrate that the Suf pathway has a fundamental role in maintaining the apicoplast organelle in addition to any role in isoprenoid biosynthesis.

## Results

### The *Plasmodium falciparum* genome encodes two distinct FeS cluster synthesis pathways

Bioinformatic studies suggest that the genomes of *Plasmodium* spp. encode both Isc and Suf proteins, including candidate cysteine desulfurases [20], [31], [32], [33], [34]. In most eukaryotes the cysteine desulfurases of the Isc and Suf pathways act in complex with the effector proteins Isd11 and SufE, respectively. SufE is essential for Suf FeS cluster synthesis in *E. coli* [15], [16], [35], but was originally thought to be absent from malaria parasites [32], [34]. More recent bioinformatic studies identified a potential *sufE* gene [30], [36] and a candidate *isd11* gene [33], [37]. We used the PATS [38], PlasmoAP [39], and PlasMit [40] algorithms to predict the subcellular localization of *P. falciparum* Suf and Isc pathway proteins (**Table 1**). Most of the Suf pathway proteins were predicted to be apicoplast localized while the Isc proteins were predicted to be mitochondrial. In other systems, however, there is precedence for dual localization and crosstalk between components of Isc and Suf pathways. In *Arabidopsis*, SufE is dually localized to chloroplasts and mitochondria and activates both the Isc and Suf cysteine desulfurases [18]. In *E. coli*, SufE serves only the Suf pathway; however, the cluster transfer proteins are interchangeable between the pathways. SufA can rescue an IscA knockout, demonstrating that SufA can interact with the rest of the Isc pathway proteins; likewise, IscA can interact with the Suf machinery [35]. In *S. cerevisiae*, IscS has been localized to the mitochondria as well as the nucleus where it has a poorly defined but essential role [41]. In order to understand how the *P. falciparum* Suf and Isc pathways are partitioned in the parasite, we localized the IscS and SufS cysteine desulfurases and their effector proteins Isd11 and SufE in blood stage parasites.

**Table 1:** The *P. falciparum* genome encodes two complete FeS cluster synthesis pathways.

			PATS^a^	PATS^a^	PlasmoAP^a^	PlasMit^a^
Pathway	Function	Gene Name (PlasmoDB)	Score	Decision	Decision	Jury (%)
**Suf**						
	Cysteine desulfurase	SufS (PF3D7_0716600)	.947	api	api	Non-mito (99)
	Cysteine desulfurase partner	SufE (PF3D7_0206100)	.977	api	api	Non-mito (99)
	Scaffold	SufB (PFC10_API0012)^b^				
		SufC (PF3D7_1413500)	.862	api	Not api	Mito (91)
		SufD (PF3D7_1103400)	.93	api	api	Non-mito (99)
	Transfer	SufU (PF3D7_0921400)	.972	api	api	Non-mito (99)
		SufA (PF3D7_0522700)	.951	api	api	Non-mito (99)
**Isc**						
	Cysteine desulfurase	IscS (PF3D7_0727200)	.046	Not api	Not api	Mito (91)
	Cysteine desulfurase partner	Isd11 (PF3D7_1311000)	.025	Not api	Not api	Mito (91)
	Scaffold	IscU1 (PF3D7_1454500)	.049	Not api	Not api	Mito (91)
		IscU2 (PF3D7_0930900)	.468	Not api	Not api	Mito (91)
	Transfer	IscA1 (PF3D7_0207200)	.119	Not api	Not api	Mito (91)
		IscA2 (PF3D7_0322500)	.055	Not api	Not api	Mito (91)

a Subcellular localization was predicted with PATS [38], PlasmoAP [39], and PlasMit [40] algorithms.

b Encoded on the apicoplast genome.

### SufS and SufE are exclusively located in the apicoplast

**Figure 2:**
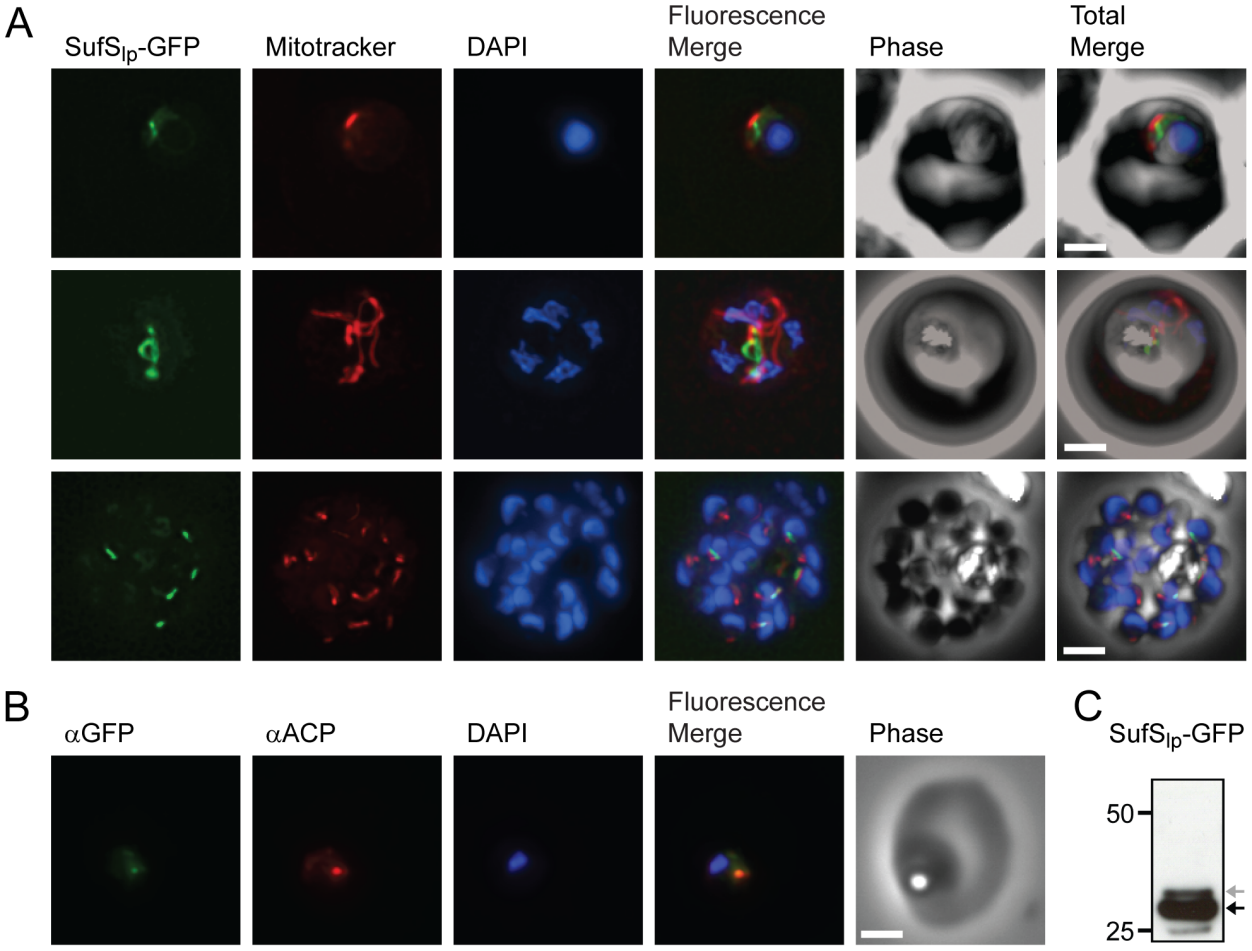
Subcellular localization of SufS to the apicoplast. **A**) Epifluorescent images of live *P. falciparum* erythrocytic-stage parasites expressing GFP fused to the SufS leader peptide (SufS_lp_-GFP). The parasites were stained with mitotracker to identify mitochondria and DAPI to identify nuclei. Image z-stacks were deconvolved and then presented as a single combined image. Scale bar = 2 µm. GFP fluorescence localizes to an elongated organelle distinct from the mitochondrion in late ring (top panel), late trophozoite or early schizont (middle), and schizont (bottom) stage parasites. **B**) Co-localization of SufS_lp_-GFP with endogenous ACP. An antibody specific for GFP co-localizes with αACP antibodies, demonstrating apicoplast localization. **C**) Proteolytic processing of SufS_lp_-GFP. An αGFP western blot identifies the mature form (black arrow) as well as a minor population of unprocessed protein (grey arrow) prior to cleavage of the apicoplast transit peptide.

We localized the SufS and SufE proteins in *P. falciparum* by expressing protein constructs fused to a C-terminal green fluorescent protein (GFP) tag. For SufS, the leader peptide (SufS_lp_) consisting of the first 59 amino acids was appended to GFP, since this region was predicted by the PATS algorithm [38] to contain the organellar targeting peptide. The mycobacteriophage Bxb1 integrase method was used to generate parasite strains with a single copy of SufS_lp_-GFP integrated into a specific recombination site in the *P. falciparum* genome [42], [43]. Live fluorescence microscopy demonstrated the presence of GFP fluorescence in an elongated organelle distinct from the parasite mitochondrion, which is typical of apicoplast morphology (**Figure 2A**). To verify localization to the apicoplast, we performed immunofluorescence analysis using antibodies against the apicoplast marker acyl carrier protein (ACP) (**Figures 2B** **and S1**). We also visualized the processing of this fusion protein upon import into the apicoplast by western blot using an antibody against GFP (**Figure 2C**). There was a small amount of unprocessed SufS_lp_-GFP while the majority of the fusion protein ran as a smaller processed species consistent with a cleavage event that occurs upon import into the apicoplast [44].

**Figure 3:**
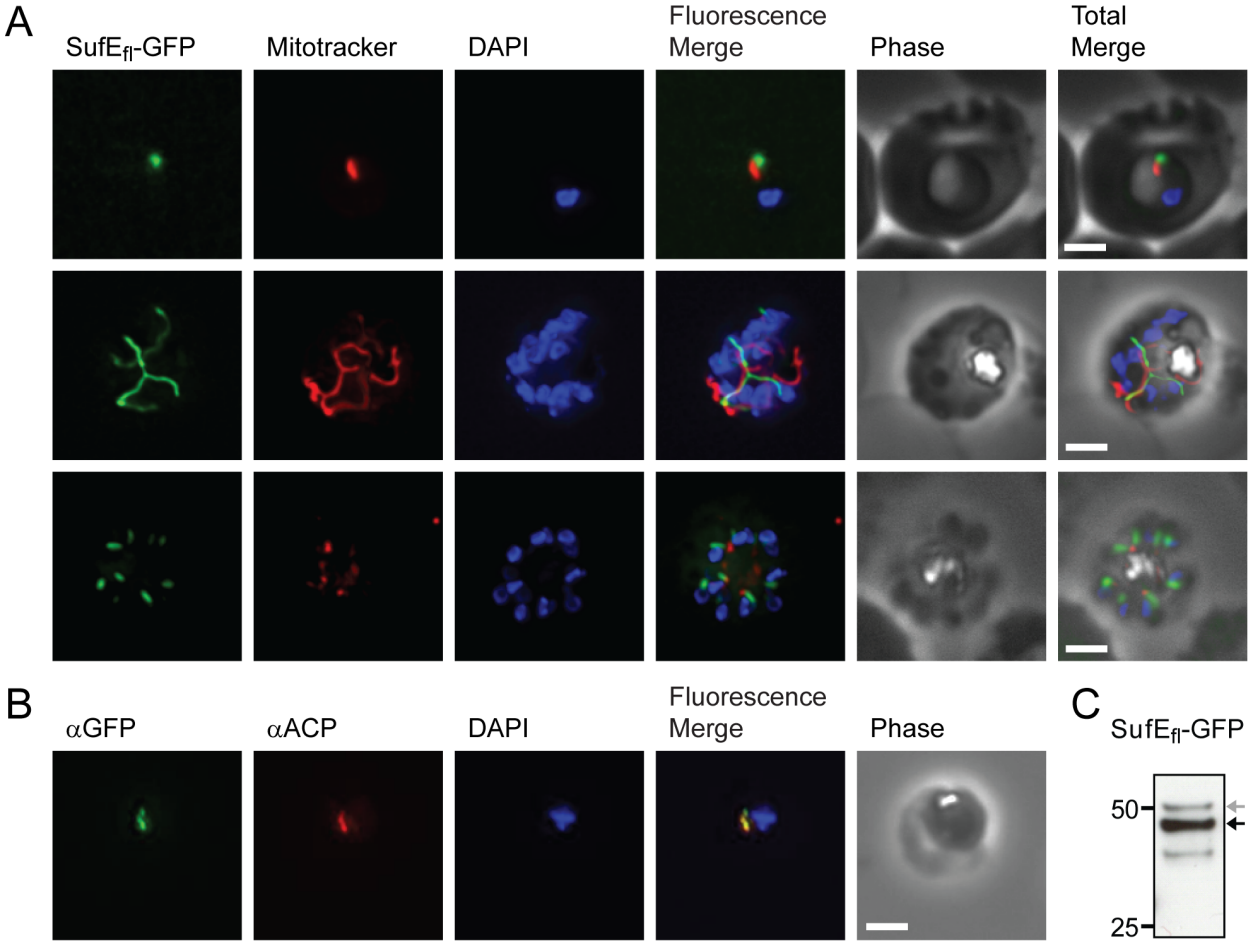
Subcellular localization of SufE to the apicoplast. **A**) Epifluorescent images of live *P. falciparum* erythrocytic-stage parasites expressing GFP fused to full length SufE (SufE_fl_-GFP). The parasites were stained with mitotracker to identify mitochondria and DAPI to identify nuclei. Image z-stacks were deconvolved and then presented as a single combined image. Scale bar = 2 µm. GFP fluorescence localizes to an elongated organelle distinct from the mitochondrion in late ring (top panel), late trophozoite or early schizont (middle), and schizont (bottom) stage parasites. **B**) Co-localization of SufE_fl_-GFP with endogenous ACP. An antibody specific for GFP co-localized with αACP antibodies, demonstrating apicoplast localization. **C**) Proteolytic processing of SufE_fl_-GFP. An αGFP western blot identifies the mature form (black arrow) as well as a minor population of unprocessed protein (grey arrow) prior to cleavage of the apicoplast transit peptide.

Full length SufE (SufE_fl_-GFP) could not be expressed in *P. falciparum* when driven by the strong calmodulin (CaM) promoter. Therefore, we used the lower strength ribosomal L2 protein (RL2) promoter [45]. SufE_fl_-GFP parasites displayed the same ramified pattern as SufS expressing parasites by live microscopy (**Figure 3A**). Detection by immunofluorescence demonstrated co-localization of SufE_fl_-GFP with the ACP apicoplast marker (**Figures 3B** **and S2**). As observed for SufS, SufE_fl_-GFP also appears to be processed, consistent with import into the apicoplast (**Figure 3C**). These results demonstrate that SufS and SufE are localized to the apicoplast of erythrocytic stage *P. falciparum* and that SufE does not appear to be dually localized as observed in *A. thaliana* [18].

### *P. falciparum* SufS is an active cysteine desulfurase that complements the loss of *E. coli* SufS

**Figure 4:**
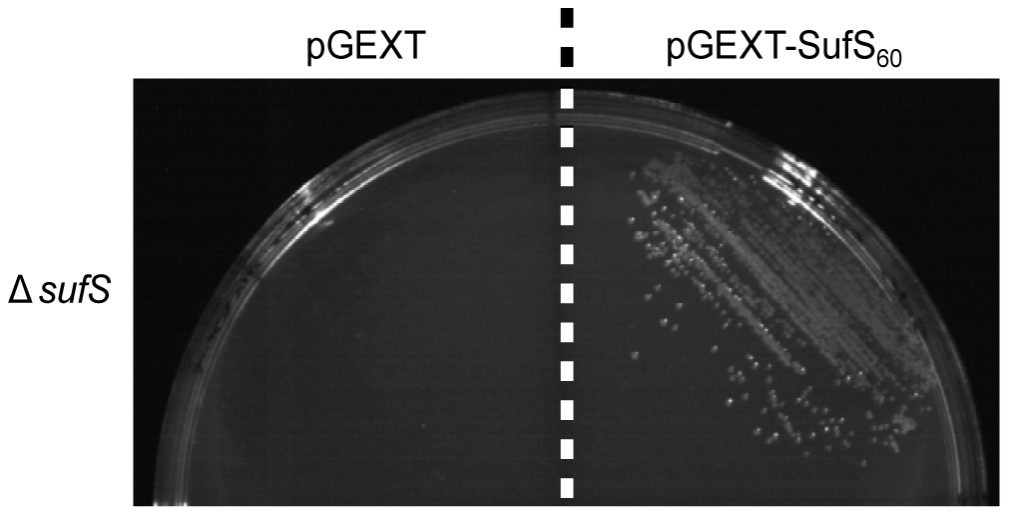
SufS cysteine desulfurase activity. SufS-null *E. coli (ΔsufS)* transformed with either empty pGEXT vector (left) or pGEXT-SufS_60_ (right) were cultured in the presence of 100 µM 2,2′-dipyridyl (an iron chelator). Under conditions of iron starvation, the *P. falciparum* gene rescues the Δ*sufS* growth phenotype, demonstrating that SufS functions as a cysteine desulfurase.

In *E. coli*, the Isc and Suf pathways are partially redundant; deletions of essential elements of either pathway result in conditional lethality while deletion of both pathways is lethal [35]. *E. coli* deficient in the Suf pathway are more sensitive to iron starvation and oxidative stress than wild type or Isc deficient strains [35]. We used the iron starvation phenotype to test the cysteine desulfurase activity of SufS in *E. coli*. Δ*sufS E. coli* transformed with the mature (processed) form of SufS (pGEXT-SufS_60_) were able to grow in the presence of an iron chelator (2,2′-dipyridyl) while Δ*sufS E. coli* transformed with empty vector (pGEXT) were unable to grow (**Figure 4**). Thus, SufS can complement the loss of *Ec*SufS, demonstrating that the parasite protein has cysteine desulfurase activity. This result also demonstrates that SufS is able to participate in an active *E. coli* Suf complex, even though mature SufS is only 30% identical to *Ec*SufS.

### IscS and Isd11 are exclusively located in the mitochondrion

**Figure 5:**
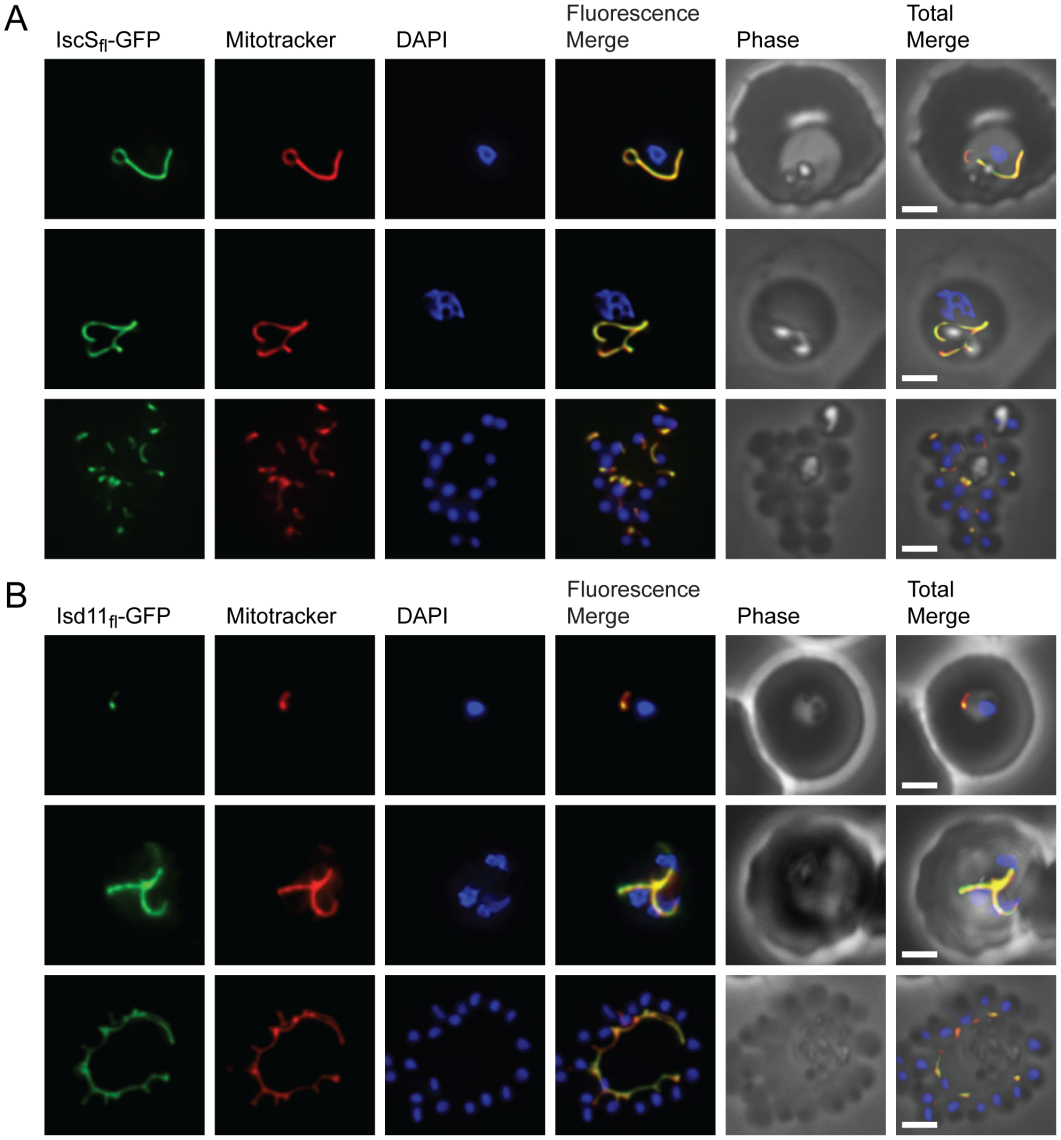
Subcellular localization of IscS and Isd11 to the mitochondrion of *P. falciparum*. **A**) Epifluorescent images of live *P. falciparum* erythrocytic-stage parasites expressing GFP fused to full-length IscS (IscS_fl_-GFP). The parasites were stained with mitotracker to identify mitochondria and DAPI to identify nuclei. Image z-stacks were deconvolved and then presented as a single combined image. Scale bar = 2 µm. **B**) Epifluorescent images similar to those in (**A**) of parasites expressing GFP fused to full length Isd11 (Isd11_fl_-GFP). IscS and Isd11 co-localize with mitotracker in all erythrocytic stages: late ring (top panels), late trophozoite or early schizont (middle panels), and schizont (lower panels) stage parasites.

We next wanted to know whether SufS is the only cysteine desulfurase that functions in the apicoplast. We localized IscS, the only other candidate cysteine desulfurase in malaria parasites, and its effector protein Isd11, using the same strategy described above for the Suf proteins. A full-length IscS construct (IscS_fl_) fused to GFP co-localized with mitotracker in live fluorescence microscopy (**Figure 5A**). Additionally, the 35 amino acid leader peptide of IscS (IscS_lp_, as predicted by PlasMit [40]) is sufficient to target GFP to the mitochondrion (**Figure S3**). We used the same integration strategy to localize a full-length construct of Isd11 (Isd11_fl_), which in yeast is necessary to activate IscS. Live fluorescence showed complete co-localization with mitotracker indicating the exclusive presence of Isd11 in the mitochondrion (**Figure 5B**). Thus, both IscS and Isd11 are mitochondrial and there is no evidence of additional nuclear localization of IscS as reported for *S. cerevisiae* IscS [41]. Taken together, these results suggest that SufS and SufE are solely responsible for sulfur acquisition for FeS synthesis in the apicoplast and we next attempted to determine whether this activity is essential in blood stage malaria parasites.

### SufE(C154S)-HA is trafficked to the apicoplast but fails to elicit a dominant negative phenotype

A conserved cysteine (at residue 51) in *E. coli* SufE is required for rapid transfer of sulfur from SufS to the SufBCD complex (**Figure 1**), and mutant SufE (C51S) binds to SufS and the SufBCD complex in a nonproductive manner [15], [16]. We attempted to interfere with iron-sulfur cluster synthesis and downstream metabolic pathways by generating an overexpression construct of *P. falciparum* SufE with the equivalent cysteine substituted with serine, SufE(C154S)-HA. We were able to select parasites expressing SufE(C154S)-HA (**Figure S4A**) in the presence of 200 µM IPP, however this parasite line was not dependent on IPP for growth (**Figure S4B**). Western blot analysis identified two protein bands for SufE(C154S)-HA, consistent with processing of the apicoplast leader peptide, and immunofluorescence showed that the protein co-localized with the apicoplast marker ACP (**Figure S4C**). Thus, SufE(C154S)-HA was expressed and properly trafficked to the apicoplast organelle, but ultimately failed to interfere with apicoplast metabolism enough to make these parasites dependent on IPP supplementation. Although SufE(C154S)-HA failed to act as a dominant negative mutant, this construct helps to confirm the apicoplast localization observed with SufE-GFP in **Figure 3**.

### SufC dominant negative parasites rely on IPP for growth

**Figure 6:**
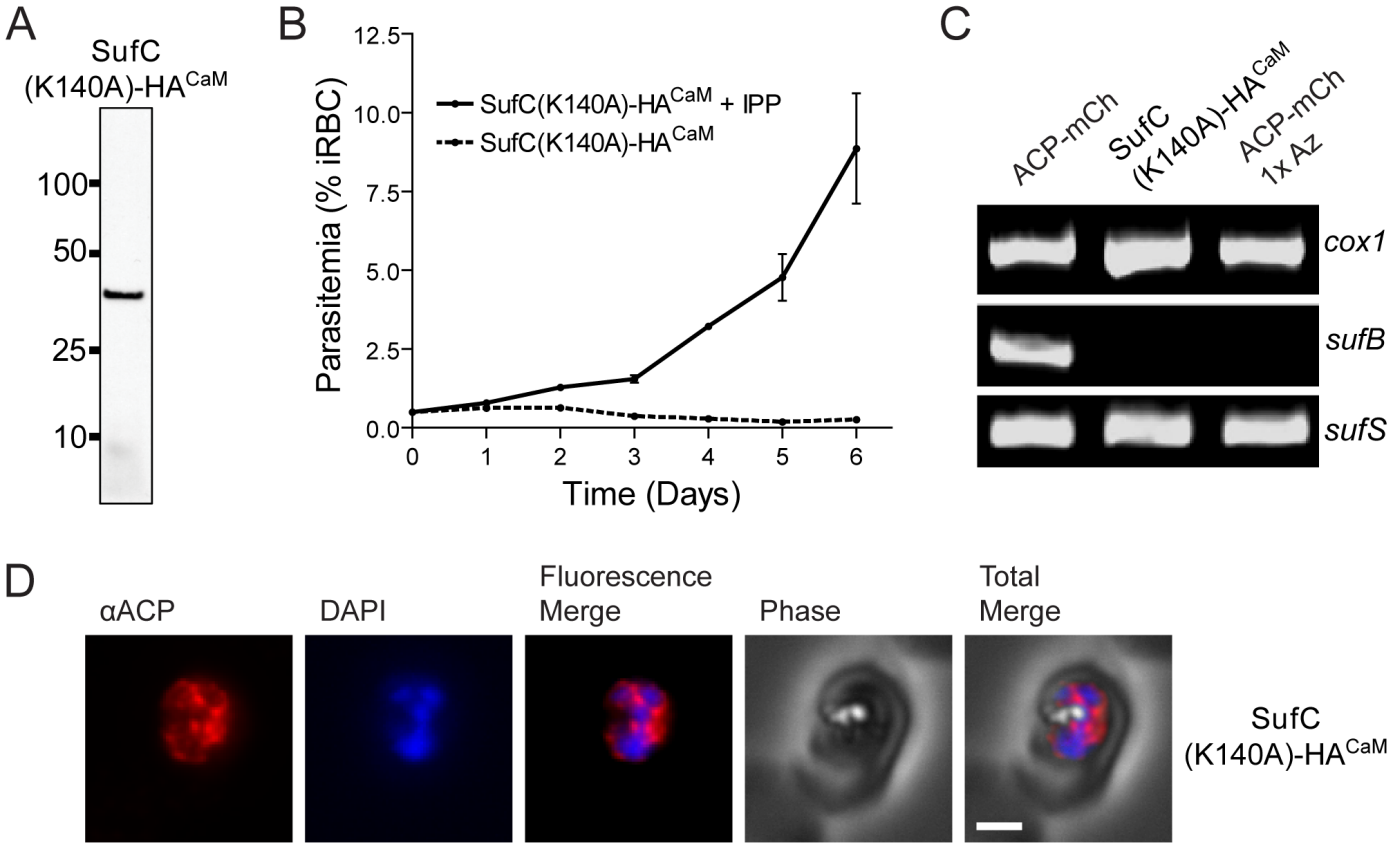
Chemical bypass of the SufC(K140A)-HA^CaM^ dominant negative construct. **A**) Expression of SufC(K140A)-HA^CaM^. An αHA western blot confirms that parasites transfected with the dominant negative construct and selected in the presence of IPP express the SufC(K140A)-HA^CaM^ construct. **B**) IPP growth dependence of SufC(K140A)-HA^CaM^ parasites. SufC(K140A)-HA^CaM^ expressing parasites survive when supplemented with IPP (solid line) but fail to grow when IPP is withdrawn (dashed line). Error bars represent SEM of triplicate measurements. Similar results were observed in two other independent experiments. **C**) Loss of the apicoplast genome. Genes from the mitochondrial genome (*cox1*), the apicoplast genome (*sufB*) and the nuclear genome (*sufS*) were amplified from the dominant negative parasite line as well as two lines expressing ACP-mCherry (ACP-mCh). The apicoplast gene *sufB* is present in untreated parasites, but is not present in azithromycin-treated (1× Az) parasites and the dominant negative SufC(K140A)-HA^CaM^ line. **D**) Localization of endogenous ACP in SufC(K140A)-HA^CaM^ parasites. Antibodies specific for the apicoplast marker ACP were used to visualize punctate vesicles present throughout the cell. Image z-stacks were deconvolved and then presented as a single combined image. Scale bar = 2 µm.

We designed another dominant negative mutant based on the recent finding that an active site lysine in *E. coli* SufC is required for ATPase activity and for accumulation of iron on the SufBCD assembly complex [46]. We generated a construct of *P. falciparum* SufC driven by the calmodulin promoter (CaM) with the active site lysine substituted with alanine, SufC(K140A)-HA^CaM^. We were unable to select parasites expressing the SufC(K140A)-HA^CaM^ construct, suggesting that expression of this construct is toxic. To bypass this toxicity, we then transfected parasites with the SufC(K140A)-HA^CaM^ construct in the presence of 200 µM IPP and were able to select transgenic parasites. Western blot analysis showed a single band consistent with the expected molecular weight of our dominant negative construct (**Figure 6A**). However, unlike the apicoplast proteins shown in **Figures 2C**, **3C** **and S4A**, there was no indication of apicoplast leader peptide processing with the SufC(K140A)-HA^CaM^ construct. Presumably, this construct was initially targeted to the apicoplast, but over time its expression led to apicoplast dysfunction and a loss of apicoplast leader peptide processing. Consistent with the effects of a dominant negative likely disrupting isoprenoid biosynthesis, these parasites were only able to grow when supplied with IPP (**Figure 6B**).

### SufC dominant negative parasites no longer contain an apicoplast organelle

We next examined the condition of the apicoplast in dominant negative parasites. We generated a control parasite line expressing the apicoplast targeting peptide of the acyl carrier protein (ACP) fused to the red fluorescent protein mCherry. Parasites expressing ACP-mCherry (ACP-mCh) were cultured for six days with IPP in the presence or absence of 100 nM azithromycin (1× IC_50_). Azithromycin treatment is known to result in loss of the apicoplast organelle [28], [47]. Parasites treated with azithromycin (1× Az) were compared to untreated parasites and dominant negative parasites using a PCR assay. We amplified genes from the apicoplast genome (*sufB*), the mitochondrial genome (*cox1*) and the nuclear genome (*sufS*). All parasite lines maintained their mitochondrial and nuclear genomes. However, in contrast to the wild type parasites, the azithromycin-treated parasites and the SufC(K140A)-HA^CaM^ dominant negative parasites no longer contained *sufB*, indicating that both strains had lost the apicoplast genome (**Figure 6C**). The localization of apicoplast marker ACP in dominant negative parasites, and the SufC(K140A)-HA protein itself, were examined by immunofluorescence and found to be present in multiple foci spread throughout the cell rather than in a single apicoplast organelle (**Figures 6D** **and S5**). The same phenotype was observed when the apicoplast was chemically disrupted [28], confirming that the apicoplast had been similarly disrupted in the dominant negative parasites.

### The SufC(K140A)-HA phenotype is specifically caused by the K140A mutation

**Figure 7:**
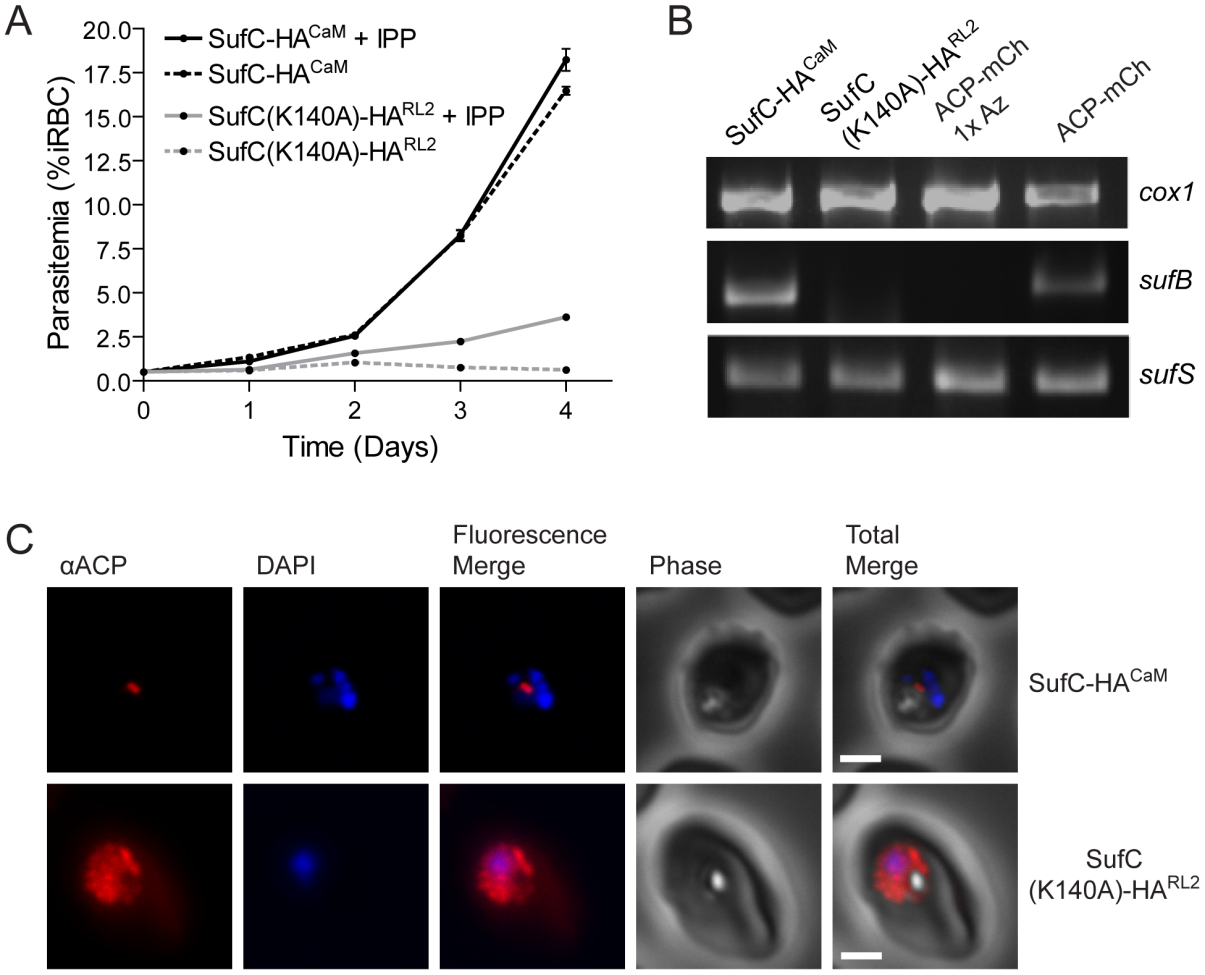
The SufC K140A mutation is responsible for the loss of apicoplast phenotype. **A**) IPP growth dependence of parasites expressing wild type SufC-HA^CaM^ driven by the calmodulin promoter and parasites expressing SufC(K140A)-HA^RL2^ driven by the weaker RL2 promoter. Parasites expressing SufC-HA^CaM^ grow both in the presence (solid black line) and absence (dashed black line) of exogenous IPP. SufC(K140A)-HA^RL2^ expressing parasites survive when supplemented with IPP (solid grey line) but fail to grow when IPP is withdrawn (dashed grey line). Error bars represent SEM of triplicate measurements. Similar results were observed in two other independent experiments. **B**) Loss of the apicoplast genome. Genes from the mitochondrial genome (*cox1*), the apicoplast genome (*sufB*) and the nuclear genome (*sufS*) were amplified from the SufC-HA^CaM^ parasites, the SufC(K140A)-HA^RL2^ line, as well as two lines expressing ACP-mCherry (ACP-mCh). The apicoplast gene *sufB* is present in the SufC-HA^CaM^ line and in the untreated parasites (ACP-mCh), but is not present in azithromycin-treated (1× Az) parasites or the SufC(K140A)-HA^RL2^ line. **C**) Localization of endogenous ACP in SufC-HA^CaM^ and SufC(K140A)-HA^RL2^ parasites. Antibodies specific for the apicoplast marker ACP were used to visualize the apicoplast in the SufC-HA^CaM^ line. The presence of multiple puncta in the SufC(K140A)-HA^RL2^ line are consistent with loss of the organelle. Image z-stacks were deconvolved and then presented as a single combined image. Scale bar = 2 µm.

It is possible that high level expression of SufC(K140A)-HA^CaM^ could interfere with secretory pathway function, leading to general toxicity and loss of the apicoplast. To test this, we generated a parasite line expressing SufC(K140A)-HA from the weaker strength RL2 (ribosomal protein L2) promoter [45], SufC(K140A)-HA^RL2^. In contrast to the CaM driven construct presented in **Figure 6A**, we were unable to detect the protein by western blot, even when the blot was loaded with ten-fold more SufC(K140A)-HA^RL2^ parasite material. Despite the lower expression level, this mutant line was also dependent on continuous supplementation with IPP for growth (**Figure 7A**) and PCR analysis indicated that these parasites no longer contained the apicoplast *sufB* gene (**Figure 7B**). Furthermore, immunofluorescence analysis of this line showed localization of ACP to multiple puncta spread throughout the cell similar to that seen with the strong promoter (**Figure 7C**). Thus, even when expressed at a lower level, the SufC(K140A)-HA^RL2^ dominant negative construct still causes the loss of the apicoplast organelle.

We next generated a parasite line expressing wild type SufC-HA driven by the CaM promoter, SufC-HA^CaM^, to test whether overexpression of this construct would lead to loss of the apicoplast. Unlike the SufC(K140A)-HA^CaM^ line shown in **Figure 6**, the SufC-HA^CaM^ line is not dependent on IPP for growth, has not lost the *sufB* gene, and appears to contain a single intact apicoplast organelle (**Figure 7**). The SufC-HA^CaM^ construct is expressed in this parasite line and co-localizes with ACP in the apicoplast organelle (**Figure S6**). Notably, the SufC-HA^CaM^ construct is processed in a manner consistent with apicoplast import (**Figure S6A**) where as the SufC(K140A)-HA^CaM^ construct is not (**Figure 6A**). Similarly, endogenous ACP protein is processed in the SufC-HA^CaM^ line, but not in the dominant negative line (**Figure S7**). Taken together, these data demonstrate that the toxicity of the dominant negative construct is not due to the expression level or the presence of the HA tag, but rather depends solely on the K140A mutation.

### Inhibition of the MEP isoprenoid biosynthesis pathway does not result in loss of the apicoplast

**Figure 8:**
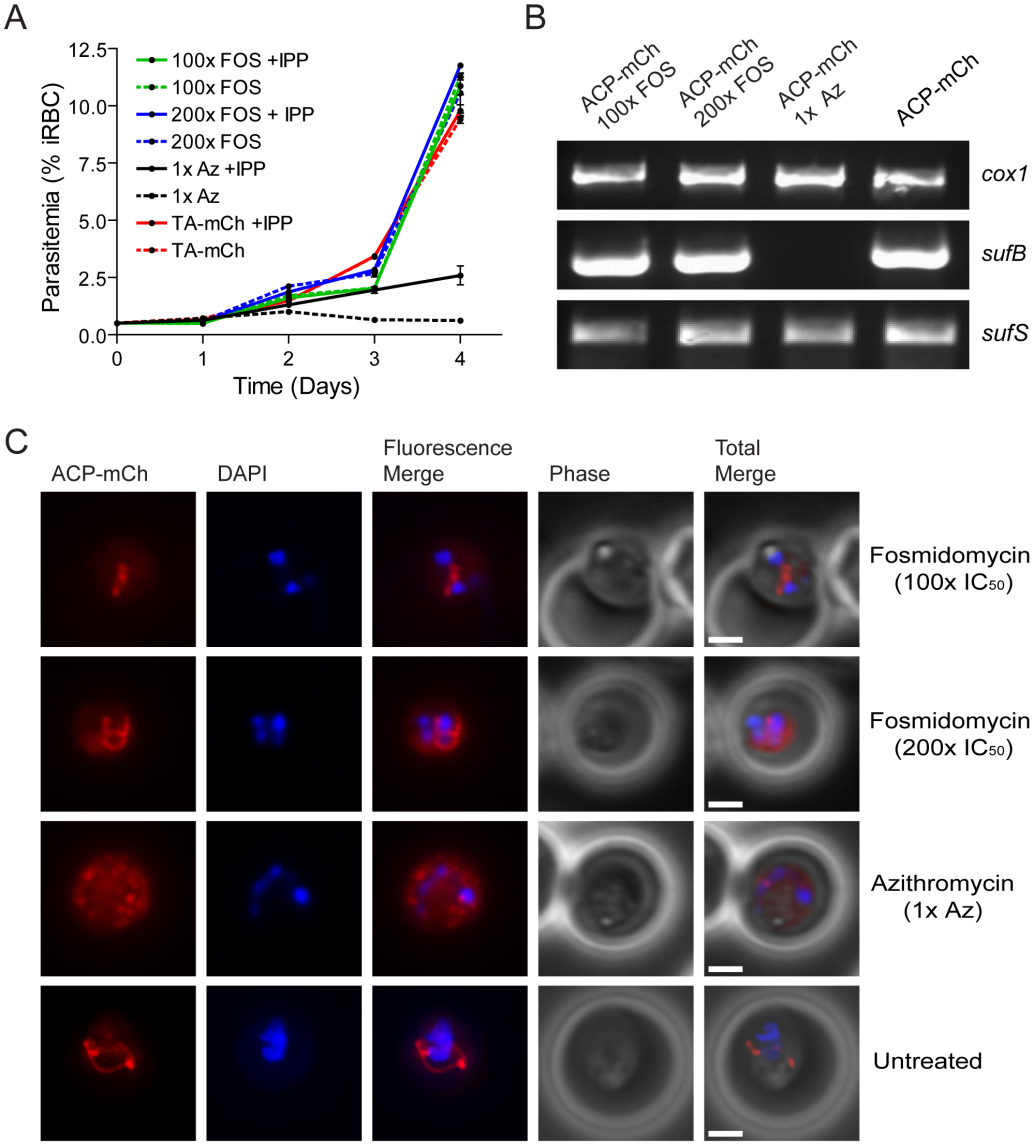
Disruption of the isoprenoid pathway does not lead to apicoplast loss. **A**) IPP growth dependence of azithromycin (Az) and fosmidomycin (FOS) treated parasites. Parasites expressing apicoplast targeted mCherry protein (ACP-mCh) were treated with 100× IC_50_ fosmidomycin (green) and 200× IC_50_ fosmidomycin (blue) for six days. Both lines grew in the presence of exogenous IPP (solid lines) and its absence (dashed lines), similar to untreated parasites (red). ACP-mCh parasites treated with 1× IC_50_ azithromycin for six days survived when supplemented with IPP (solid black line) but failed to grow when IPP was withdrawn (dashed black line). **B**) Loss of the apicoplast genome. Genes from the mitochondrial genome (*cox1*), the apicoplast genome (*sufB*) and the nuclear genome (*sufS*) were amplified from the four ACP-mCh lines. The apicoplast gene *sufB* is present in parasites treated with fosmidomycin (100× FOS and 200× FOS), but is not present in azithromycin-treated parasites (1× Az). **C**) Loss of apicoplast organelle morphology. Epifluorescent images of live parasites grown in the presence of IPP are shown with nuclei stained with DAPI. The apicoplast as marked by mCherry fluorescence no longer appears as a branched continuous organelle in azithromycin-treated parasites while the apicoplast remains intact in untreated and fosmidomycin-treated parasites. Image z-stacks were deconvolved and then presented as a single combined image. Scale bar = 2 µm.

Isoprenoids produced by the MEP pathway are the only metabolites produced in the apicoplast that are required outside of the organelle during the erythrocytic stages [28]. It is not known, however, whether the MEP pathway is required for maintenance of the apicoplast itself. To test this, we treated parasites with either azithromycin, to target all apicoplast functions, or fosmidomycin, which specifically targets the MEP pathway. We inhibited the MEP pathway in the presence of IPP by treating ACP-mCh parasites with 50 µM fosmidomycin (100× IC_50_), 100 µM fosmidomycin (200× IC_50_), 100 nM azithromycin (1× IC_50_), or no drug for six days. In subsequent growth experiments, only the azithromycin-treated parasites were dependent on IPP for growth (**Figure 8A**). Consistent with this growth phenotype, these parasites also lacked the apicoplast gene *sufB* (**Figure 8B**). These results indicate that treatment with azithromycin leads to loss of the apicoplast organelle while treatment with fosmidomycin does not. The ACP-mCherry produced by fosmidomycin-treated parasites and untreated control parasites is trafficked to a single branched organelle consistent with normal apicoplast morphology (**Figure 8C**). By contrast, parasites treated in parallel with azithromycin contain ACP-mCherry in multiple foci throughout the cell (**Figure 8C**). Thus, inhibition of the MEP pathway with fosmidomycin does not lead to loss of the apicoplast organelle. The dominant negative disruption of the Suf pathway results in similar molecular and cellular phenotypes as the general disruption of the apicoplast by azithromycin and not the specific inhibition of the MEP pathway by fosmidomycin (**Figures 6** **and** **7**). These results suggest that in addition to providing FeS clusters for isoprenoid biosynthesis enzymes, the Suf pathway also plays a role in the maintenance of the apicoplast organelle.

## Discussion

**Figure 9:**
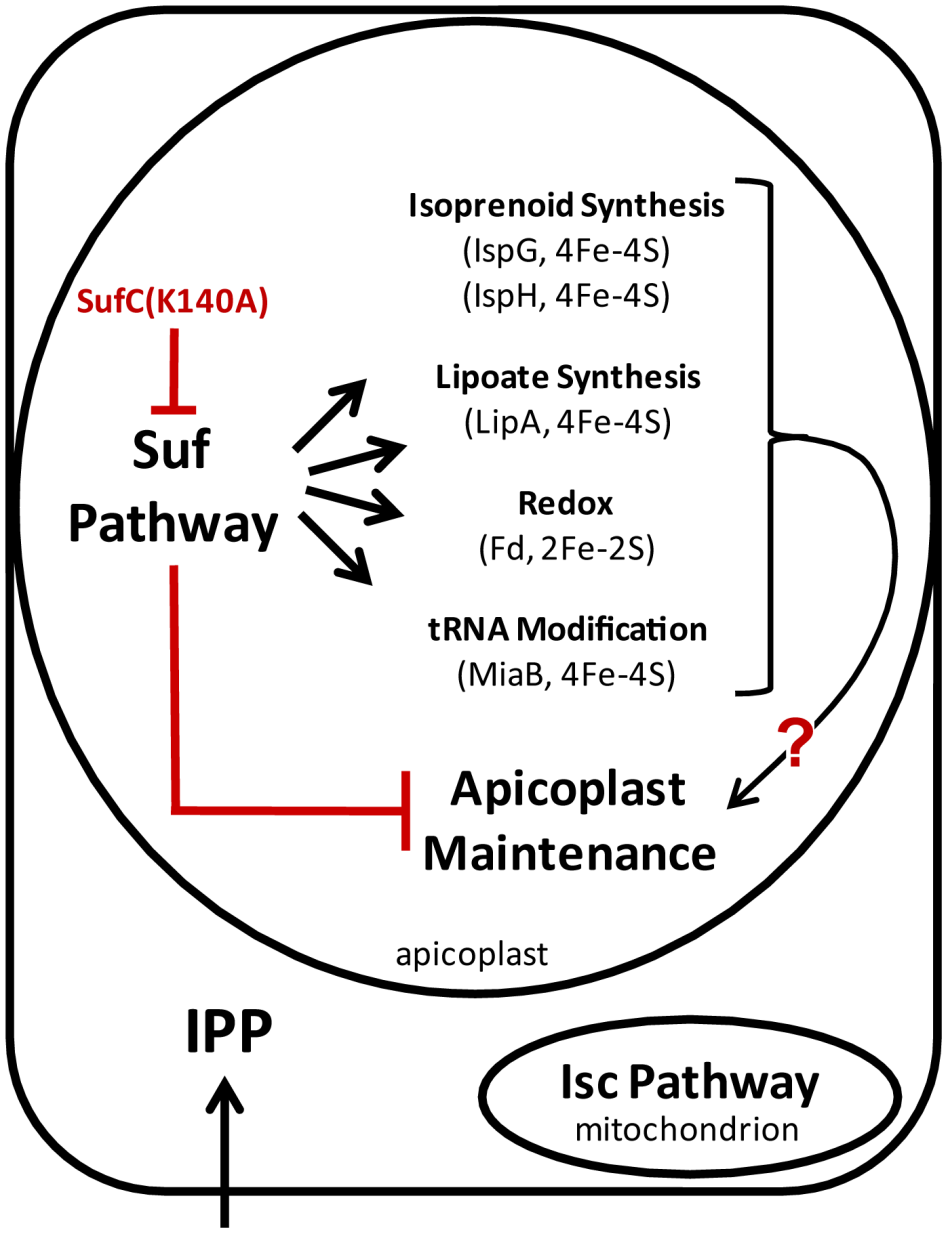
Disruption of the Suf pathway leads to apicoplast loss. The Isc pathway is located in the mitochondrion while the Suf pathway is located in the apicoplast. The exclusive division of the two pathways between the two endosymbiont organelles suggests that the FeS-dependent proteins the apicoplast rely solely on the Suf FeS cluster synthesis pathway. When the Suf pathway was disrupted by expressing the dominant negative probe SufC(K140A) the apicoplast was no longer maintained. One or more of the FeS cluster dependent proteins present in the apicoplast could be required for maintenance of the organelle.

In the apicoplast of *Plasmodium falciparum* there are several pathways that are predicted to rely on FeS cluster cofactors (**Figure 9**), and one of these pathways is known to be essential for erythrocytic stage growth. An early step in MEP isoprenoid synthesis is the target for the antimalarial fosmidomycin [29] which is currently being evaluated in human trials as a partner drug with piperaquine. Recently, it was shown that supplementing parasites with isopentenyl pyrophosphate (IPP, one of the two final products of the MEP pathway) rescues sensitivity to antibiotics targeting apicoplast maintenance (e.g. chloramphenicol, clindamycin, doxycycline), demonstrating that isoprenoid synthesis is essential for blood stage parasite growth [28]. Antibiotic-treated parasites no longer contain an intact apicoplast or the organellar genome, however, these abnormalities should not affect the expression of the MEP pathway proteins. All of the enzymes in the MEP pathway are nuclear encoded and should still be produced under conditions in which the apicoplast is disrupted by antibiotic treatment. This is certainly true for the nuclear encoded apicoplast protein ACP, which is still produced regardless of whether the apicoplast is disrupted (**Figures 6** **,** **7** **and S7**). Unlike ACP, the enzymes that catalyze the penultimate and final steps of isoprenoid synthesis (IspG and IspH, respectively) should both contain FeS clusters [22], [27]. As described below, these clusters should not be available in parasites that lack an apicoplast.

SufB is one of the few non-housekeeping genes encoded in the apicoplast genome. In other systems, SufB plays an essential role in FeS cluster assembly and is the scaffold on which the clusters are built [35], [46]. When apicoplast maintenance is disrupted, SufB, and thereby FeS cluster synthesis, should be lost; this would then lead to disruption of the MEP pathway. Consistent with these expectations, we found that disruption of the Suf pathway with the SufC(K140A)-HA^CaM^ dominant negative mutant was toxic to blood stage malaria parasites. Parasites were only viable if supplemented with IPP, indicating that disruption of the Suf pathway ultimately leads to loss of the MEP isoprenoid biosynthesis pathway (**Figure 6**). Thus, the Suf pathway supports the MEP pathway and is essential for the survival of blood stage malaria parasites.

In addition to the MEP pathway, the apicoplast of malaria parasites harbors a type II fatty acid synthesis (FASII) pathway which is essential for liver stage development [48], [49]. The FASII pathway consumes acetyl-CoA [50] which is produced by the apicoplast-localized pyruvate dehydrogenase (PDH) enzyme complex [51]. Like the FASII pathway, a complete PDH complex (composed of four proteins) is essential during liver stage parasite development [52]. PDH is modified with the protein cofactor lipoate [53] which should be required for enzymatic activity. The synthesis of lipoate in the apicoplast is catalyzed by lipoate synthase (LipA), which we have shown contains 4Fe-4S clusters (**Figure S8**). These FeS clusters not only need to be synthesized, but they probably also need to be continuously repaired. One of the FeS clusters in *E. coli* LipA is destroyed every time lipoate is formed, making turnover of LipA dependent on replacing this FeS cluster [54]. Thus, FeS cluster synthesis in the apicoplast should ultimately be required for lipoate synthesis, PDH activity, and the function of the FASII pathway known to be critical for liver stage development in rodent and human malaria parasite species.

In organisms expressing both an Isc and a Suf pathway, such as *E. coli*, the Isc pathway acts as the default FeS synthesis pathway while the Suf pathway is expressed under conditions of prolonged oxidative stress and iron starvation [35]. It has been suggested that the Suf pathway is more efficient than the Isc pathway under conditions of oxidative stress [55]; this would be an attractive characteristic of the FeS cluster synthesis pathway expressed in oxygen producing compartments such as the plant chloroplast or the ancestral photosynthetic apicoplast [6], [56]. However, the modern apicoplast appears to maintain a reducing environment and is highly resistant to oxidative stress [57]. This protective environment may enhance the activity of enzymes sensitive to oxygen, such as LipA (**Figure S8**), but it is not clear whether the parasite Suf pathway retains the tolerance to oxidative stress conditions displayed by its orthologs in plants and bacteria.

FeS clusters are synthesized by ancient, highly conserved pathways, at least one of which is found in all organisms [58]. We have confirmed the presence of the Suf pathway in the *P. falciparum* apicoplast (**Figures 2**, **3** **, S1 and S2**) and demonstrated the activity of the cysteine desulfurase SufS, the first enzyme in the pathway (**Figure 4**). In 2003, another group localized IscS as a test of a transfection method [59]. They fused GFP to what was at the time predicted to be the first 135 amino acids of IscS, however, the amino-terminus of the current gene model differs from the sequence used in that study. We repeated the localization using the current gene model which aligns more closely with eukaryotic IscS sequences. *P. falciparum* IscS and Isd11 both localized exclusively to the mitochondrion (**Figures 5** **and S3**). The subcellular partitioning of the Isc and Suf pathways demonstrates that they function independently of each other, and are likely both essential for erythrocytic stage parasite growth. The same general pattern of organellar partitioning of the Isc and Suf pathways is observed in the only other plastid-containing organism in which both pathway components have been localized, *A. thaliana* [6]. *P. falciparum* appears to differ from *A. thaliana*, however, in that we observe SufE solely in the apicoplast while one of the *Arabidopsis* SufE homologs appears to be dually localized between the chloroplasts and the mitochondria and has been shown to activate mitochondrial *At*IscS [18]. *At*Isd11 is only 18% identical to Isd11 from *P. falciparum* and *At*IscS lacks the extended amino terminus of IscS present in *P. falciparum* and *S. cerevisiae*. There may be functional differences between these IscS homologs that affect their ability to be stimulated by effector proteins.

FeS cluster modified proteins in the *P. falciparum* mitochondrion are involved in redox regulation, metabolism, and participate in the electron transport chain. Complex III (cytochrome bc1) is the target of the antimalarial atovaquone, which prevents binding of reduced ubiquinone and also blocks electron transfer from the Rieske type 2Fe-2S cluster, implying that the Isc pathway is essential for blood stage parasite growth [60]. This makes the Isc pathway an attractive drug target, however it is closely related to the host Isc pathway. Closer study of the Isc pathway found in parasites may identify exploitable differences between mitochondrial FeS cluster synthesis in the parasite and in the human host.

The Suf pathway is not found in humans, and the work presented here shows that it is required for the maintenance of the apicoplast organelle. If the Suf pathway was only needed to activate certain MEP enzymes, we would expect disruption of the Suf pathway to have similar effects as inhibition of the MEP pathway. This, however, was not the case. As shown in **Figure 8**, inhibition of the MEP pathway by the specific inhibitor fosmidomycin does not lead to dependence on IPP for growth, loss of the apicoplast gene *sufB*, or observable changes in organelle morphology. In contrast to fosmidomycin treatment, disruption of the Suf pathway with the dominant negative mutant SufC(K140A)-HA^CaM^ results in loss of the apicoplast organelle. Dominant negative parasites depend on IPP for growth, have lost the *sufB* gene, and no longer contain an intact apicoplast organelle (**Figures 6**).

One possible explanation for this broader phenotype is that high level expression of SufC(K140A)-HA^CaM^ interfered with secretory pathway function, leading to general toxicity. This seems unlikely, however, since these parasites still traffic ACP into punctate foci in the cell (**Figures 6C** **and S5**), consistent with the membrane-bound secretory vesicles observed by Yeh and coworkers [28]. To address this issue, we generated two additional parasite lines. The first expressed the same dominant negative mutant driven by the lower strength RL2 promoter. This parasite line, SufC(K140A)-HA^RL2^, displayed the same loss of apicoplast phenotype, demonstrating the potency of the dominant negative SufC(K140A) mutation (**Figure 7**). We also generated a parasite line expressing a wild type construct of SufC driven by the strong calmodulin promoter. This SufC-HA^CaM^ construct differs from the toxic dominant negative SufC(K140A)-HA^CaM^ construct by a single amino acid, yet had none of the molecular and cellular phenotypes associated with loss of the apicoplast organelle (**Figures 7** **and S6**). Thus, the K140A point mutation is solely responsible for disrupting apicoplast metabolism leading to loss of the organelle.

How does the dominant negative mutant interfere with apicoplast metabolism? SufC is known to bind to SufB [30] and presumably forms the SufBCD iron-sulfur cluster assembly complex observed in other organisms (**Figure 1**). The SufC(K140A) mutant was designed to form a nonproductive complex with endogenous SufB and SufD, thereby limiting the availability of these proteins for cluster assembly. The dominant negative mutant should decrease cluster synthesis, but it could also affect iron homeostasis in the apicoplast, a phenomenon that is difficult to study since organellar iron import and storage mechanisms are not known. We attempted to interfere with the Suf pathway at an earlier step (sulfur acquisition) with the SufE(C154S) mutant, but this construct did not have a dominant negative phenotype, even when overexpressed with the strong calmodulin promoter (**Figure S4**). It may be that sulfur acquisition is not the rate limiting step in the parasite Suf pathway or that the SufE mutant does not interact with other Suf proteins as observed in the *E. coli* system.

The most likely effect of the SufC(K140A) dominant negative mutant is inactivation of apicoplast FeS proteins. Known and predicted FeS proteins are shown in **Figure 9**, including four FeS enzymes (LipA, IspH, IspG and MiaB) and ferredoxin (Fd). Are any of these proteins likely to be required for apicoplast maintenance during blood stage parasite growth? As described above, LipA is responsible for lipoylating the PDH and ultimately supporting fatty acid biosynthesis in the apicoplast. Since components of the FASII pathway and subunits of the PDH complex (albeit not the lipoylated E2 subunit) have been successfully deleted in blood stage malaria parasites [48], [49], [52], LipA is presumed to be similarly dispensable and not required for apicoplast maintenance. Although IspH and IspG should be essential for isoprenoid biosynthesis in blood stage parasites, loss of these enzymes should have the same effect as inhibition with fosmidomycin. As shown in **Figure 8**, inhibition of isoprenoid biosynthesis does not result in loss of the apicoplast organelle.

This result also suggests that the final FeS enzyme, MiaB, is not required for apicoplast maintenance. MiaB presumably functions in conjunction with an upstream enzyme, MiaA, in the maturation of tRNAs. MiaA has not been studied in malaria parasites, but in most eukaryotes and bacteria this enzyme transfers isopentenyl groups to a specific adenosine base in the anticodon loop of certain tRNAs [61], [62]. MiaB is a methylthiolase that further modifies the isopentenyladenosine tRNA base with a CH_3_S group [63], [64]. If *P. falciparum* MiaB functions in an analogous way, then its activity depends on isoprenoid biosynthesis, a pathway that we have shown is not required for apicoplast maintenance. Importantly, MiaA enzymes use the MEP pathway product DMAPP (dimethylallyl pyrophosphate) as the source of isopentenyl groups and would not be able to use the IPP that we supply in our parasite culture conditions unless there is an IPP/DMAPP isomerase present. Thus, based on their predicted activities (these enzymes could have additional noncanonical activities), these four FeS enzymes do not appear to be good candidates to explain why disrupting FeS cluster synthesis leads to loss of the apicoplast organelle.

Among the predicted apicoplast FeS proteins in **Figure 9**, ferredoxin stands out as the most integral to apicoplast function. *P. falciparum* Fd contains a 2Fe-2S cluster and has been shown to act as an electron donor to IspH [27]. Other apicoplast pathways may also depend on Fd, since it is predicted to be the preferred electron transfer partner for the other apicoplast FeS enzymes (LipA, IspG and MiaB) and may be required to provide reducing equivalents during certain steps of FeS cluster biosynthesis [36], [65]. Because of its role in FeS synthesis, reduced Fd metalation could have an exaggerated effect by further limiting the production of its own FeS clusters. Even if Fd is required for FeS synthesis, it still does not provide an explanation for how the apicoplast is lost since the downstream FeS enzymes do not have obvious roles in apicoplast maintenance. Loss of the organelle may instead be linked to how redox balance is maintained in the apicoplast. Fd in conjunction with its associated reductase, ferredoxin-NADP^+^-reductase (FNR), is the only known redox system in the apicoplast [66]. Perturbation of the Fd/FNR system could lead to increased sensitivity to oxidative stress, as observed in other systems [67]. Since the apicoplast is known to be a highly reducing environment [57], failure of this protective system could lead to oxidative damage, particularly of the organellar DNA, and subsequent loss of the organelle. Regardless of the mechanism, it is clear that Suf pathway dysfunction results in a disruption of apicoplast maintenance. Since the enzymes which comprise the Suf pathway are distinct from anything found in the human host, they are attractive targets for inhibition. The Suf pathway appears to lie at the root of apicoplast metabolic function and inhibition of the pathway should block the growth of blood stage and liver stage malaria parasites.

## Materials and Methods

### Generation of *P. falciparum* transfection constructs

The genes in this study were amplified from gDNA or cDNA prepared from blood stage *P. falciparum* Dd2 strain parasites and inserted into the pLN-GFP transfection plasmid described by Nkrumah and coworkers [42]. In some cases, the calmodulin (CaM) promoter of pLN-GFP was substituted with the weaker strength ribosomal L2 protein (RL2) promoter [45], and in other cases the GFP tag was removed or replaced with mCherry (mCh) or a hemagglutinin tag (HA).

The *iscS* gene (PF3D7_0727200) was amplified from gDNA with primers IscS.AvrII.F and IscS.fl.BsiWI.R and inserted into pRL2-GFP, generating plasmid pRL2-IscS_fl_-GFP (see **Table S1** for primer sequences). Nucleotides encoding the 35 amino acid IscS leader peptide (IscS_lp_) were amplified from pRL2-IscS_fl_-GFP vector using primers IscS.AvrII.F and IscS.35.BsiWI.R and ligated into pLN-GFP to generate pLN-IscS_lp_-GFP. The in-frame intron in *isd11* (PF3D7_1311000) was confirmed by amplifying this gene from cDNA with primers Isd11.AvrII.F and Isd11.BsiWI.R and inserting into pRL2-GFP, generating plasmid pRL2-Isd11_fl_-GFP. The *sufS* gene (PF3D7_0716600) was amplified from cDNA using the primers SufS.TOPO.F and SufS.TOPO.R and ligated into cloning vector pET100/D-TOPO (Invitrogen). Nucleotides encoding the leader peptide of SufS were amplified using the primers SufS.AvrII.F and SufS.59.BsiWI.R and ligated into pLN-GFP, generating plasmid pLN-SufS_lp_-GFP. Amplification of *sufE* (PF3D7_0206100) from cDNA confirmed the four exon gene model, but consistently resulted in a frame-shifted amplicon. Gene synthesis (GeneArt) was used to generate the *sufE* gene flanked by *Avr*II and *Bsi*WI endonuclease sites which were used to subclone into the pRL2-GFP transfection plasmid generating pRL2-SufE_fl_-GFP.

A transfection vector was created to express mCherry red fluorescent protein in the apicoplast organelle. The gene encoding mCherry was amplified with primers mCh.BsiWI.F and mCh.AflII.R and inserted into the pLN-TP-ACP-GFP vector described by Gallagher *et al.* [68]. The resulting transfection vector, pLN-TP-ACP-mCh, encodes mCherry instead of GFP. Constucts SufE(C154S), SufC (PF3D7_1413500) and SufC(K140A) were synthezised (GeneArt) with flanking *Avr*II and *Bsi*WI sites and inserted into a pLN plasmid modified to have a carboxy-terminal single HA tag, generating pLN-SufE(C154S)-HA, pLN-SufC-HA and pLN-SufC(K140A)-HA. The SufC(K140A)-HA coding region was digested from this plasmid with *Avr*II and *Bsi*WI and inserted into pRL2 to generate pRL2-SufC(K140A)-HA.

### *P. falciparum* transfection and maintenance

*P. falciparum* transfections were performed using the Bxb1 mycobacteriophage integrase system in Dd2 strain parasites containing the attB recombination site [42] in combination with a red blood cell (RBC) preloading technique [43]. Infected red blood cells (iRBC) were first observed between 11 and 27 days after beginning selection with 2.5 µg/mL blasticidin. Insertion of the transgene at the *attB* site was confirmed by PCR using the primers P1, P2, P3, and P4 (**Table S1**) as described by Spalding *et al.* [43]. Genomic DNA from each integrated parasite line was purified and used to verify the transgene sequence with primers GFP.R or pLN.790.R and either RL2.F or CaM.F, as appropriate (**Table S1**).

Parasites were maintained in human red blood cell culture at 2% hematocrit using the general method described by Trager and Jensen [69]. Briefly, blood stage parasites were cultured in RPMI 1640 supplemented with 10% human serum, 28 mM NaCO_3_H, 25 mM HEPES, and 0.09 mM hypoxanthine. Cultures were gassed with 92% N_2_, 3% O_2_, 5% CO_2_ and incubated in sealed 75 cm^2^ flasks at 37°C. For the chemical bypass experiments, 0.5 ml or 1 ml parasite cultures were maintained in 24 or 48 well plates and supplemented during daily feedings with 200 µM isopentenyl pyrophosphate (Sigma).

### Epifluorescent microscopy and western analysis

Parasite cultures with a parasitemia between 2% and 15% were incubated for 30 minutes at 37°C with 12.5 nM mitotracker CMX-Ros (Invitrogen) and 1 µg/mL 4′, 6-diamidino-2-phenylindole (DAPI). Cells were washed three times for 5 minutes at 37°C with RPMI or PBS and then sealed on a slide for observation on a Nikon Eclipse 90i equipped with an automated z-stage. A series of images spanning 4 µm were acquired with 0.2 µm spacing and images were deconvolved with VOLOCITY software (PerkinElmer) to report a single combined z -stack image.

Parasites were fixed and permeabilized for immunofluorescence studies. Live parasites were mixed with 4% paraformaldehyde and 0.0075% glutaraldehyde in PBS and placed on poly-lysine coated glass slides for 30 minutes at room temperature. The slides were then incubated with 1% Triton X-100 in PBS for 10 minutes and then washed three times for 5 minutes with PBS. Sodium borohydride (0.1 g/L in PBS) was used to reduce any remaining unreacted aldehydes followed by three more 5 minutes washes in PBS. The slides were then blocked with 3% bovine serum albumin for an hour and then probed with the appropriate primary antibodies [1∶500 rabbit or rat αACP [57], 1∶50 Living Colors mouse αGFP JL-8 (Clontech), or 1∶50 rat αHA mAb 3F10 (Roche)]. Slides were washed three times for 5 minutes with PBS, and then incubated with the appropriate secondary antibodies [1∶3,000 goat αRabbit IgG *Alexa Fluor* 594, 1∶1,000 goat αMouse IgG *Alexa Fluor* 488 (Invitrogen), or 1∶1,000 goat αRat IgG *Alexa Fluor* 488 (Invitrogen)] for one hour at room temperature. The slides were washed three times for five minutes with PBS, then mounted with Prolong Gold antifade reagent with DAPI (Invitrogen).

Expression of SufS and SufE in transgenic parasites was verified by western blot. Host RBCs from 5 mL cultures at 5–15% parasitemia were permeabilized with 0.2% saponin in PBS for 5 min on ice and then washed repeatedly in PBS until the supernatant was clear. Purified parasites were then lysed in gel loading buffer and parasite proteins were resolved on a NuPage 4–12% Bis-Tris reducing gel (Invitrogen) and transferred onto nitrocellulose. The nitrocellulose membrane was blocked for at least one hour with 5% milk in PBS and probed overnight at 4°C with 1∶5,000 Living Colors mouse αGFP JL-8 (Clonetech) in 1% milk. The membrane was washed with PBS three times and probed with 1∶20,000 sheep αMouse IgG horseradish peroxidase (HRP) secondary antibody (GE Healthcare) for at least one hour at room temperature. After three additional washes, the blot was visualized with SuperSignal West Pico detection solution (Thermo Scientific) and exposed to film.

### Generation of *E. coli* expression constructs

All constructs expressed in *E. coli* were cloned into the pGEXT vector which expresses the parasite proteins fused to a cleavable amino terminal glutathione-s-transferase (GST) tag [70]. Mature *lipA* (encoding residues 89 to 415 of PF3D7_1344600) was amplified from cDNA using *Pfu* polymerase and primers LipA.EcoRI.F and LipA.PstI.R (**Table S1**). This amplicon was digested with *Pst*I and *Eco*RI and ligated into vector pMALcHT [71]. Primers LipA.BamHI.F and LipA.EcoRI.R (**Table S1**) were used to subclone LipA, generating plasmid pGEXT-LipA_89_. A construct of mature *sufS* (encoding residues 60 to 546 of PF3D7_0716600) was amplified from vector pET100/D-TOPO (described above) using primers SufS.BamHI.F and SufS.EcoRI.R (**Table S1**), generating plasmid pGEXT-SufS_60_.

### Complementation of *E. coli sufS*

*E. coli* containing a deletion of *sufS* (*ΔsufS*, Keio collection JW1670) were transformed with either empty vector, pGEXT, or pGEXT-SufS_60_. Each strain was grown overnight at 37°C in MinE medium as modified by Allary *et al.* [53]. The overnight culture was used to plate 1 µL of 1.0 OD_600_ on MinE agar plates containing 100 µM 2,2′-dipyridyl. The plates were incubated for 48 hrs at 30°C and inspected for bacterial growth.

### Anaerobic expression and purification of LipA

BL21 Star (DE3) *E. coli* containing the pLysE plasmid were transformed with pGEXT-LipA_89_ construct produced above. In order to culture the protein in conditions of minimal oxygen, *E. coli* were grown in flat bottom flasks filled three quarters full with LB medium. When cells reached an OD_600_ of 0.6 they were induced with 0.4 mM IPTG for 10 hours at 20°C. Cells were harvested by centrifugation, flash frozen in liquid nitrogen, and stored under the liquid layer. The cell pellet was transferred to a Bactron IV (Shell Labs) anaerobic chamber flooded with 5% hydrogen, 5% carbon dioxide, and 90% nitrogen. A palladium catalyst was used to maintain <30 ppm oxygen. Cells were resuspended in anaerobic lysis buffer (20 mM Na/K phosphate [pH 7.5], 200 mM NaCl, 2 g/L lysozyme, and 1 mM phenylmethylsulfonyl fluoride [PMSF]) and incubated at room temperature until cell lysis was apparent. After lysis, 2.5 µg/mL DNase I was added and incubated for 30 minutes at room temperature. The lysate was transferred to an air tight container and centrifuged to separate the soluble and insoluble fractions. GST-LipA_89_ was purified using a 5 mL GST-Trap HP column (GE Healthcare) connected to a peristaltic pump in the anaerobic chamber.

### Characterization of dominant negative and control parasite lines

Plasmids pLN-SufE(C154S)-HA, pLN-SufC(K140A)-HA, pRL2-SufC(K140A)-HA and pLN-SufC-HA were used to generate transgenic parasite lines. As described above, these parasite lines were maintained in the presence of 200 µM IPP. Protein expression was confirmed by western blot using the methods described above with 1∶1,000 rat αHA mAb 3F10 (Roche) and 1∶20,000 goat αRat IgG horseradish peroxidase (HRP) secondary antibody (GE Healthcare) secondary antibody. Growth assays were conducted in triplicate using 24 well culture plates and initiated at a parasitemia of 0.5%. Over a six day period, parasitemia was assessed by flow cytometry using a FACSCalibur cell sorting machine (Becton Dickinson). Samples of 10 µl from each well were incubated with 10 µl of 5 µM dihydroethidium for 15 minutes at 37°C in the dark. Results were analyzed by FlowJo software (Tree Star Inc., Ashland, OR). Whole cell PCR was used to amplify representative genes from the nuclear (*sufS*), apicoplast (*sufB*), and mitochondrial (*cox1*) genomes (**Table S1**). Phusion High-Fidelity DNA Polymerase (New England BioLabs) was used in accordance with the manufacturer's directions in 25 µL reactions containing 1 µL of parasite culture. The processing of endogenous ACP was visualized by western blot using the 4% formaldehyde 0.1% glutaraldehyde fixation conditions previously described [57] to prevent ACP from diffusing out of the blot membrane. The blot was probed with 1∶5,000 rabbit αACP [57] primary and 1∶3,500 donkey αRabbit IgG horseradish peroxidase (HRP) secondary antibody (GE Healthcare).

### Generation of the inhibitor-treated ACP-mCherry lines

Parasites transfected with the pLN-TA-ACP-mCherry vector were supplemented with 200 µM IPP and treated with 100 nM azithromycin, 50 µM fosmidomycin, 100 µM fosmidomycin, or no drug for 6 days. All four ACP-mCherry (ACP-mCh) lines were then tested for IPP dependence and analyzed by live epifluorescence microscopy and whole cell PCR as descibed above.

## Supporting Information

Figure S1 **Immunofluorescence co-localization of SufS_lp_-GFP and endogenous ACP.** An antibody specific for GFP co-localized with αACP antibodies, demonstrating apicoplast localization in late ring (top panel), late trophozoite or early schizont (middle), and schizont (bottom) stage parasites. The parasites were stained with DAPI to identify nuclei. Image z-stacks were deconvolved and then presented as a single combined image. Scale bar = 2 µm.
(TIF)

Figure S2 **Immunofluorescence co-localization of SufE_fl_-GFP and endogenous ACP.** An antibody specific for GFP co-localized with αACP antibodies, demonstrating apicoplast localization in late ring (top panel), late trophozoite or early schizont (middle), and schizont (bottom) stage parasites. The parasites were stained with DAPI to identify nuclei. Image z-stacks were deconvolved and then presented as a single combined image. Scale bar = 2 µm.
(TIF)

Figure S3 **Subcellular localization of the IscS leader peptide to the mitochondrion of** ***P. falciparum*** **.** Epifluorescent images of live *P. falciparum* erythrocytic-stage parasites expressing GFP fused to the leader peptide of IscS (amino acids 1–35). The parasites were stained with mitotracker to identify mitochondria and DAPI to identify nuclei. Image z-stacks were deconvolved and then presented as a single combined image. Scale bar = 2 µm.
(TIF)

Figure S4 **Overexpression of SufE(C154S)-HA in the apicoplast.** **A**) Expression of SufE(C154S)-HA. An αHA western blot confirms the expression of the SufE(C154S)-HA construct and identifies the mature form (black arrow) as well as a minor population of unprocessed protein (grey arrow) prior to cleavage of the apicoplast transit peptide. **B**) IPP growth dependence of SufE(C154S)-HA parasites. SufE(C154S)-HA expressing parasites survive when supplemented with IPP (solid line) and when IPP is withdrawn (dashed line). Error bars represent SEM of triplicate measurements. **C**) Co-localization of SufE(C154S)-HA with endogenous ACP. Antibodies specific for the apicoplast marker ACP were used to visualize the apicoplast in blood stage parasites. Co-localization with an antibody specific for the HA tag shows that SufE(C154S)-HA is located in the apicoplast in late ring (top panel), trophozoite (middle), and early schizonts (bottom). Image z-stacks were deconvolved and then presented as a single combined image. Scale bar = 2 µm.
(TIF)

Figure S5 **Subcellular localization of ACP and SufC(K140A)-HA shows disrupted apicoplast morphology.** **A**) Localization of endogenous ACP in SufC(K140A)-HA^CaM^ parasites. Antibodies specific for the apicoplast marker ACP were used to visualize punctate vesicles in late ring (top panel), trophozoite or early schizont (middle), and schizont (bottom) stage parasites. **B**) Co-localization of SufC(K140A)-HA protein and endogenous ACP. An antibody specific for the HA tag indicates that SufC(K140A)-HA is co-localized with ACP in late ring (top panel), early schizont (middle), and schizont (bottom) stage parasites. Image z-stacks were deconvolved and then presented as a single combined image. Scale bar = 2 µm.
(TIF)

Figure S6 **Subcellular localization of wildtype SufC to the apicoplast of** ***P. falciparum*** **.** **A**) Expression of SufC-HA^CaM^. An αHA western blot confirms the expression of the SufC-HA protein and identifies the mature form (black arrow) as well as a minor population of unprocessed protein (grey arrow) prior to cleavage of the apicoplast transit peptide. **B**) Co-localization of SufC-HA protein with endogenous ACP. An antibody specific for the HA tag co-localized with αACP antibodies, demonstrating apicoplast localization in late ring (top panel), late trophozoite or early schizont (middle), and schizont (bottom) stage parasites. The parasites were stained with DAPI to identify nuclei. Image z-stacks were deconvolved and then presented as a single combined image. Scale bar = 2 µm.
(TIF)

Figure S7 **Processing of endogenous ACP.** Antibodies specific for the Acyl Carrier Protein (ACP) were used to identify the mature form (black arrow) and the unprocessed protein (grey arrow) prior to cleavage of the apicoplast transit peptide. Only the unprocessed trafficking intermediate is present in the dominant negative SufC(K140A)-HA^RL2^ parasite line, demonstrating loss of this apicoplast function.
(TIF)

Figure S8 **Purification and characterization of recombinant LipA protein.** **A**) Lipoate synthase (LipA), was expressed as a GST fusion protein and purified from *E. coli*. Analysis by SDS-PAGE shows that GST-LipA_89_ migrates close to its predicted molecular weight of 63 kDa. Additional protein bands in the GST-LipA_89_ sample (asterisk) cross-react with antibodies specific for GST and likely result from proteolytic cleavage or incomplete translation. **B**) The UV-Vis absorption spectrum for anaerobically purified GST-LipA_89_ displays a broad peak at 440 nm typical of 4Fe-4S proteins. Because the LipA clusters are highly sensitive to oxygen, the signature 4Fe-4S UV-VIS signal degrades over time when exposed to air.
(TIF)

Table S1 **DNA primers used in this study.** The annealing portions of the sequences are underlined while the endonuclease sites are marked by boldface type.
(DOC)

## References

[1] Beinert H (2000) Iron-sulfur proteins: ancient structures, still full of surprises. J Biol Inorg Chem 5: 2–15. 10766431 10.1007/s007750050002

[2] Brzoska K, Meczynska S, Kruszewski M (2006) Iron-sulfur cluster proteins: electron transfer and beyond. Acta Biochim Pol 53: 685–691. 17143336

[3] Beinert H, Holm RH, Munck E (1997) Iron-sulfur clusters: nature's modular, multipurpose structures. Science 277: 653–659. 9235882 10.1126/science.277.5326.653

[4] Kennedy C, Dean D (1992) The nifU, nifS and nifV gene products are required for activity of all three nitrogenases of Azotobacter vinelandii. Mol Gen Genet 231: 494–498. 1538703 10.1007/BF00292722

[5] Muhlenhoff U, Lill R (2000) Biogenesis of iron-sulfur proteins in eukaryotes: a novel task of mitochondria that is inherited from bacteria. Biochim Biophys Acta 1459: 370–382. 11004453 10.1016/s0005-2728(00)00174-2

[6] Balk J, Lobreaux S (2005) Biogenesis of iron-sulfur proteins in plants. Trends Plant Sci 10: 324–331. 15951221 10.1016/j.tplants.2005.05.002

[7] Pilon-Smits EA, Garifullina GF, Abdel-Ghany S, Kato S, Mihara H, et al (2002) Characterization of a NifS-like chloroplast protein from Arabidopsis. Implications for its role in sulfur and selenium metabolism. Plant Physiol 130: 1309–1318. 12427997 10.1104/pp.102.010280 PMC166651

[8] Leon S, Touraine B, Briat JF, Lobreaux S (2002) The AtNFS2 gene from Arabidopsis thaliana encodes a NifS-like plastidial cysteine desulphurase. Biochem J 366: 557–564. 12033984 10.1042/BJ20020322 PMC1222802

[9] Tsaousis AD, Ollagnier de Choudens S, Gentekaki E, Long S, Gaston D, et al (2012) Evolution of Fe/S cluster biogenesis in the anaerobic parasite Blastocystis. Proc Natl Acad Sci U S A 109: 10426–10431. 22699510 10.1073/pnas.1116067109 PMC3387072

[10] Adam AC, Bornhovd C, Prokisch H, Neupert W, Hell K (2006) The Nfs1 interacting protein Isd11 has an essential role in Fe/S cluster biogenesis in mitochondria. EMBO J 25: 174–183. 16341090 10.1038/sj.emboj.7600905 PMC1356348

[11] Wiedemann N, Urzica E, Guiard B, Muller H, Lohaus C, et al (2006) Essential role of Isd11 in mitochondrial iron-sulfur cluster synthesis on Isu scaffold proteins. EMBO J 25: 184–195. 16341089 10.1038/sj.emboj.7600906 PMC1356349

[12] Paris Z, Changmai P, Rubio MA, Zikova A, Stuart KD, et al (2010) The Fe/S cluster assembly protein Isd11 is essential for tRNA thiolation in Trypanosoma brucei. J Biol Chem 285: 22394–22402. 20442400 10.1074/jbc.M109.083774 PMC2903368

[13] Richards TA, van der Giezen M (2006) Evolution of the Isd11-IscS complex reveals a single alpha-proteobacterial endosymbiosis for all eukaryotes. Mol Biol Evol 23: 1341–1344. 16648156 10.1093/molbev/msl001

[14] Pandey A, Yoon H, Lyver ER, Dancis A, Pain D (2011) Isd11p protein activates the mitochondrial cysteine desulfurase Nfs1p protein. J Biol Chem 286: 38242–38252. 21908622 10.1074/jbc.M111.288522 PMC3207418

[15] Layer G, Gaddam SA, Ayala-Castro CN, Ollagnier-de Choudens S, Lascoux D, et al (2007) SufE transfers sulfur from SufS to SufB for iron-sulfur cluster assembly. J Biol Chem 282: 13342–13350. 17350958 10.1074/jbc.M608555200

[16] Outten FW, Wood MJ, Munoz FM, Storz G (2003) The SufE protein and the SufBCD complex enhance SufS cysteine desulfurase activity as part of a sulfur transfer pathway for Fe-S cluster assembly in Escherichia coli. J Biol Chem 278: 45713–45719. 12941942 10.1074/jbc.M308004200

[17] Loiseau L, Ollagnier-de-Choudens S, Nachin L, Fontecave M, Barras F (2003) Biogenesis of Fe-S cluster by the bacterial Suf system: SufS and SufE form a new type of cysteine desulfurase. J Biol Chem 278: 38352–38359. 12876288 10.1074/jbc.M305953200

[18] Xu XM, Moller SG (2006) AtSufE is an essential activator of plastidic and mitochondrial desulfurases in Arabidopsis. EMBO J 25: 900–909. 16437155 10.1038/sj.emboj.7600968 PMC1383551

[19] Fast NM, Kissinger JC, Roos DS, Keeling PJ (2001) Nuclear-encoded, plastid-targeted genes suggest a single common origin for apicomplexan and dinoflagellate plastids. Mol Biol Evol 18: 418–426. 11230543 10.1093/oxfordjournals.molbev.a003818

[20] Ralph SA, van Dooren GG, Waller RF, Crawford MJ, Fraunholz MJ, et al (2004) Tropical infectious diseases: metabolic maps and functions of the Plasmodium falciparum apicoplast. Nat Rev Microbiol 2: 203–216. 15083156 10.1038/nrmicro843

[21] Cicchillo RM, Lee KH, Baleanu-Gogonea C, Nesbitt NM, Krebs C, et al (2004) Escherichia coli lipoyl synthase binds two distinct [4Fe-4S] clusters per polypeptide. Biochemistry 43: 11770–11781. 15362861 10.1021/bi0488505

[22] Lee M, Grawert T, Quitterer F, Rohdich F, Eppinger J, et al (2010) Biosynthesis of isoprenoids: crystal structure of the [4Fe-4S] cluster protein IspG. J Mol Biol 404: 600–610. 20932974 10.1016/j.jmb.2010.09.050

[23] Rekittke I, Wiesner J, Rohrich R, Demmer U, Warkentin E, et al (2008) Structure of (E)-4-hydroxy-3-methyl-but-2-enyl diphosphate reductase, the terminal enzyme of the non-mevalonate pathway. J Am Chem Soc 130: 17206–17207. 19035630 10.1021/ja806668q PMC2756146

[24] Zepeck F, Grawert T, Kaiser J, Schramek N, Eisenreich W, et al (2005) Biosynthesis of isoprenoids. purification and properties of IspG protein from Escherichia coli. J Org Chem 70: 9168–9174. 16268586 10.1021/jo0510787

[25] Pierrel F, Bjork GR, Fontecave M, Atta M (2002) Enzymatic modification of tRNAs: MiaB is an iron-sulfur protein. J Biol Chem 277: 13367–13370. 11882645 10.1074/jbc.C100609200

[26] Kimata-Ariga Y, Kurisu G, Kusunoki M, Aoki S, Sato D, et al (2007) Cloning and characterization of ferredoxin and ferredoxin-NADP+ reductase from human malaria parasite. J Biochem 141: 421–428. 17251200 10.1093/jb/mvm046

[27] Rohrich RC, Englert N, Troschke K, Reichenberg A, Hintz M, et al (2005) Reconstitution of an apicoplast-localised electron transfer pathway involved in the isoprenoid biosynthesis of Plasmodium falciparum. FEBS Lett 579: 6433–6438. 16289098 10.1016/j.febslet.2005.10.037

[28] Yeh E, DeRisi JL (2011) Chemical rescue of malaria parasites lacking an apicoplast defines organelle function in blood-stage Plasmodium falciparum. PLoS Biol 9: e1001138. 21912516 10.1371/journal.pbio.1001138 PMC3166167

[29] Jomaa H, Wiesner J, Sanderbrand S, Altincicek B, Weidemeyer C, et al (1999) Inhibitors of the nonmevalonate pathway of isoprenoid biosynthesis as antimalarial drugs. Science 285: 1573–1576. 10477522 10.1126/science.285.5433.1573

[30] Kumar B, Chaubey S, Shah P, Tanveer A, Charan M, et al (2011) Interaction between sulphur mobilisation proteins SufB and SufC: evidence for an iron-sulphur cluster biogenesis pathway in the apicoplast of Plasmodium falciparum. Int J Parasitol 41: 991–999. 21722645 10.1016/j.ijpara.2011.05.006

[31] Wilson RJ (2005) Parasite plastids: approaching the endgame. Biol Rev Camb Philos Soc 80: 129–153. 15727041 10.1017/s1464793104006591

[32] Seeber F (2002) Biogenesis of iron-sulphur clusters in amitochondriate and apicomplexan protists. Int J Parasitol 32: 1207–1217. 12204220 10.1016/s0020-7519(02)00022-x

[33] van Dooren GG, Stimmler LM, McFadden GI (2006) Metabolic maps and functions of the Plasmodium mitochondrion. FEMS Microbiol Rev 30: 596–630. 16774588 10.1111/j.1574-6976.2006.00027.x

[34] Ellis KE, Clough B, Saldanha JW, Wilson RJ (2001) Nifs and Sufs in malaria. Mol Microbiol 41: 973–981. 11555280 10.1046/j.1365-2958.2001.02588.x

[35] Outten FW, Djaman O, Storz G (2004) A suf operon requirement for Fe-S cluster assembly during iron starvation in Escherichia coli. Mol Microbiol 52: 861–872. 15101990 10.1111/j.1365-2958.2004.04025.x

[36] Seeber F, Soldati-Favre D (2010) Metabolic pathways in the apicoplast of apicomplexa. Int Rev Cell Mol Biol 281: 161–228. 20460186 10.1016/S1937-6448(10)81005-6

[37] Mather MW, Henry KW, Vaidya AB (2007) Mitochondrial drug targets in apicomplexan parasites. Curr Drug Targets 8: 49–60. 17266530 10.2174/138945007779315632

[38] Zuegge J, Ralph S, Schmuker M, McFadden GI, Schneider G (2001) Deciphering apicoplast targeting signals–feature extraction from nuclear-encoded precursors of Plasmodium falciparum apicoplast proteins. Gene 280: 19–26. 11738814 10.1016/s0378-1119(01)00776-4

[39] Foth BJ, Ralph SA, Tonkin CJ, Struck NS, Fraunholz M, et al (2003) Dissecting apicoplast targeting in the malaria parasite Plasmodium falciparum. Science 299: 705–708. 12560551 10.1126/science.1078599

[40] Bender A, van Dooren GG, Ralph SA, McFadden GI, Schneider G (2003) Properties and prediction of mitochondrial transit peptides from Plasmodium falciparum. Mol Biochem Parasitol 132: 59–66. 14599665 10.1016/j.molbiopara.2003.07.001

[41] Nakai Y, Nakai M, Hayashi H, Kagamiyama H (2001) Nuclear localization of yeast Nfs1p is required for cell survival. J Biol Chem 276: 8314–8320. 11110795 10.1074/jbc.M007878200

[42] Nkrumah LJ, Muhle RA, Moura PA, Ghosh P, Hatfull GF, et al (2006) Efficient site-specific integration in Plasmodium falciparum chromosomes mediated by mycobacteriophage Bxb1 integrase. Nat Methods 3: 615–621. 16862136 10.1038/nmeth904 PMC2943413

[43] Spalding MD, Allary M, Gallagher JR, Prigge ST (2010) Validation of a modified method for Bxb1 mycobacteriophage integrase-mediated recombination in Plasmodium falciparum by localization of the H-protein of the glycine cleavage complex to the mitochondrion. Mol Biochem Parasitol 172: 156–160. 20403390 10.1016/j.molbiopara.2010.04.005 PMC2875341

[44] van Dooren GG, Su V, D'Ombrain MC, McFadden GI (2002) Processing of an apicoplast leader sequence in Plasmodium falciparum and the identification of a putative leader cleavage enzyme. J Biol Chem 277: 23612–23619. 11976331 10.1074/jbc.M201748200

[45] Balabaskaran Nina P, Morrisey JM, Ganesan SM, Ke H, Pershing AM, et al (2011) ATP synthase complex of Plasmodium falciparum: dimeric assembly in mitochondrial membranes and resistance to genetic disruption. J Biol Chem 286: 41312–41322. 21984828 10.1074/jbc.M111.290973 PMC3308843

[46] Saini A, Mapolelo DT, Chahal HK, Johnson MK, Outten FW (2010) SufD and SufC ATPase activity are required for iron acquisition during in vivo Fe-S cluster formation on SufB. Biochemistry 49: 9402–9412. 20857974 10.1021/bi1011546 PMC3004146

[47] Dahl EL, Rosenthal PJ (2007) Multiple antibiotics exert delayed effects against the Plasmodium falciparum apicoplast. Antimicrob Agents Chemother 51: 3485–3490. 17698630 10.1128/AAC.00527-07 PMC2043295

[48] Vaughan AM, O'Neill MT, Tarun AS, Camargo N, Phuong TM, et al (2009) Type II fatty acid synthesis is essential only for malaria parasite late liver stage development. Cell Microbiol 11: 506–520. 19068099 10.1111/j.1462-5822.2008.01270.x PMC2688669

[49] Yu M, Kumar TR, Nkrumah LJ, Coppi A, Retzlaff S, et al (2008) The fatty acid biosynthesis enzyme FabI plays a key role in the development of liver-stage malarial parasites. Cell Host Microbe 4: 567–578. 19064257 10.1016/j.chom.2008.11.001 PMC2646117

[50] Prigge ST, He X, Gerena L, Waters NC, Reynolds KA (2003) The initiating steps of a type II fatty acid synthase in Plasmodium falciparum are catalyzed by pfACP, pfMCAT, and pfKASIII. Biochemistry 42: 1160–1169. 12549938 10.1021/bi026847k

[51] Foth BJ, Stimmler LM, Handman E, Crabb BS, Hodder AN, et al (2005) The malaria parasite Plasmodium falciparum has only one pyruvate dehydrogenase complex, which is located in the apicoplast. Mol Microbiol 55: 39–53. 15612915 10.1111/j.1365-2958.2004.04407.x

[52] Pei Y, Tarun AS, Vaughan AM, Herman RW, Soliman JM, et al (2010) Plasmodium pyruvate dehydrogenase activity is only essential for the parasite's progression from liver infection to blood infection. Mol Microbiol 75: 957–971. 20487290 10.1111/j.1365-2958.2009.07034.x

[53] Allary M, Lu JZ, Zhu L, Prigge ST (2007) Scavenging of the cofactor lipoate is essential for the survival of the malaria parasite Plasmodium falciparum. Mol Microbiol 63: 1331–1344. 17244193 10.1111/j.1365-2958.2007.05592.x PMC2796473

[54] Cicchillo RM, Booker SJ (2005) Mechanistic investigations of lipoic acid biosynthesis in Escherichia coli: both sulfur atoms in lipoic acid are contributed by the same lipoyl synthase polypeptide. J Am Chem Soc 127: 2860–2861. 15740115 10.1021/ja042428u

[55] Ayala-Castro C, Saini A, Outten FW (2008) Fe-S cluster assembly pathways in bacteria. Microbiol Mol Biol Rev 72: 110–125 table of contents. 18322036 10.1128/MMBR.00034-07 PMC2268281

[56] McFadden GI (2011) The apicoplast. Protoplasma 248: 641–650. 21165662 10.1007/s00709-010-0250-5

[57] Gallagher JR, Prigge ST (2010) Plasmodium falciparum acyl carrier protein crystal structures in disulfide-linked and reduced states and their prevalence during blood stage growth. Proteins 78: 575–588. 19768685 10.1002/prot.22582 PMC2805782

[58] Dellibovi-Ragheb TA, Gisselberg JE, Prigge ST (2013) Parasites FeS up: iron-sulfur cluster biogenesis in eukaryotic pathogens. PLoS Pathog 9: e1003227. 23592980 10.1371/journal.ppat.1003227 PMC3617024

[59] Sato S, Rangachari K, Wilson RJ (2003) Targeting GFP to the malarial mitochondrion. Mol Biochem Parasitol 130: 155–158. 12946854 10.1016/s0166-6851(03)00166-x

[60] Mather MW, Darrouzet E, Valkova-Valchanova M, Cooley JW, McIntosh MT, et al (2005) Uncovering the molecular mode of action of the antimalarial drug atovaquone using a bacterial system. J Biol Chem 280: 27458–27465. 15917236 10.1074/jbc.M502319200 PMC1421511

[61] Lamichhane TN, Blewett NH, Maraia RJ (2011) Plasticity and diversity of tRNA anticodon determinants of substrate recognition by eukaryotic A37 isopentenyltransferases. Rna-a Publication of the Rna Society 17: 1846–1857. 10.1261/rna.2628611 PMC3185917 21873461

[62] Persson BC, Esberg B, Olafsson O, Bjork GR (1994) Synthesis and Function of Isopentenyl Adenosine Derivatives in Transfer-Rna. Biochimie 76: 1152–1160. 7748950 10.1016/0300-9084(94)90044-2

[63] Esberg B, Leung HCE, Tsui HCT, Bjork GR, Winkler ME (1999) Identification of the miaB gene, involved in methylthiolation of isopentenylated A37 derivatives in the tRNA of Salmonella typhimurium and Escherichia coli. Journal of Bacteriology 181: 7256–7265. 10572129 10.1128/jb.181.23.7256-7265.1999 PMC103688

[64] Hernandez HL, Pierrel F, Elleingand E, Garcia-Serres R, Huynh BH, et al (2007) MiaB, a bifunctional radical-S-adenosylmethionine enzyme involved in the thiolation and methylation of tRNA, contains two essential [4Fe-4S] clusters. Biochemistry 46: 5140–5147. 17407324 10.1021/bi7000449

[65] Seeber F, Aliverti A, Zanetti G (2005) The plant-type ferredoxin-NADP(+) reductase/ferrodoxin redox system as a possible drug target against apicomplexan human parasites. Current Pharmaceutical Design 11: 3159–3172. 16178751 10.2174/1381612054864957

[66] Vollmer M, Thomsen N, Wiek S, Seeber F (2001) Apicomplexan parasites possess distinct nuclear-encoded, but apicoplast-localized, plant-type ferredoxin-NADP(+) reductase and ferredoxin. Journal of Biological Chemistry 276: 5483–5490. 11056177 10.1074/jbc.M009452200

[67] Krapp AR, Rodriguez RE, Poli HO, Paladini DH, Palatnik JF, et al (2002) The flavoenzyme ferredoxin (flavodoxin)-NADP(H) reductase modulates NADP(H) homeostasis during the soxRS response of Escherichia coli. Journal of Bacteriology 184: 1474–1480. 11844783 10.1128/JB.184.5.1474-1480.2002 PMC134851

[68] Gallagher JR, Matthews KA, Prigge ST (2011) Plasmodium falciparum apicoplast transit peptides are unstructured in vitro and during apicoplast import. Traffic 12: 1124–1138. 21668595 10.1111/j.1600-0854.2011.01232.x PMC3629917

[69] Trager W, Jensen JB (1997) Continuous culture of Plasmodium falciparum: its impact on malaria research. Int J Parasitol 27: 989–1006. 9363481 10.1016/s0020-7519(97)00080-5

[70] Delli-Bovi TA, Spalding MD, Prigge ST (2010) Overexpression of biotin synthase and biotin ligase is required for efficient generation of sulfur-35 labeled biotin in E. coli. BMC Biotechnol 10: 73. 20937134 10.1186/1472-6750-10-73 PMC2964542

[71] Muench SP, Rafferty JB, McLeod R, Rice DW, Prigge ST (2003) Expression, purification and crystallization of the Plasmodium falciparum enoyl reductase. Acta Crystallogr D Biol Crystallogr 59: 1246–1248. 12832774 10.1107/s0907444903008813

