# Supplementary figures and images for: The Suf Iron-Sulfur Cluster Synthesis Pathway Is Required for Apicoplast Maintenance in Malaria Parasites
Source: PLoS Pathog. 2013 Sep 26;9(9):e1003655. doi: 10.1371/journal.ppat.1003655 (PMC3784473; doi:10.1371/journal.ppat.1003655)

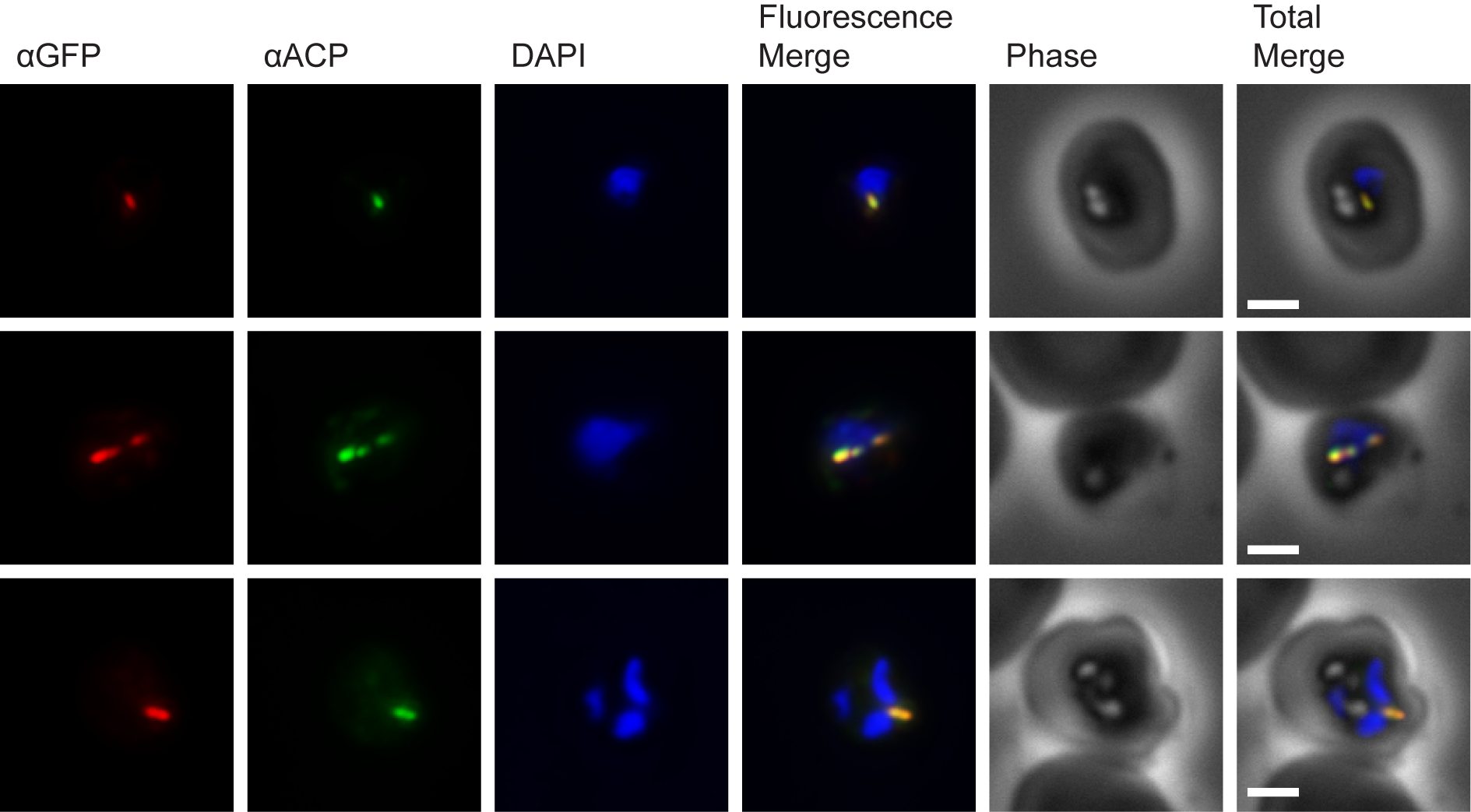

Supplement: Figure S1 — Immunofluorescence co-localization of SufSlp-GFP and endogenous ACP. An antibody specific for GFP co-localized with αACP antibodies, demonstrating apicoplast localization in late ring (top panel), late trophozoite or early schizont (middle), and schizont (bottom) stage parasites. The parasites were stained with DAPI to identify nuclei. Image z-stacks were deconvolved and then presented as a single combined image. Scale bar = 2 µm. (TIF) [file ppat.1003655.s001.tif]

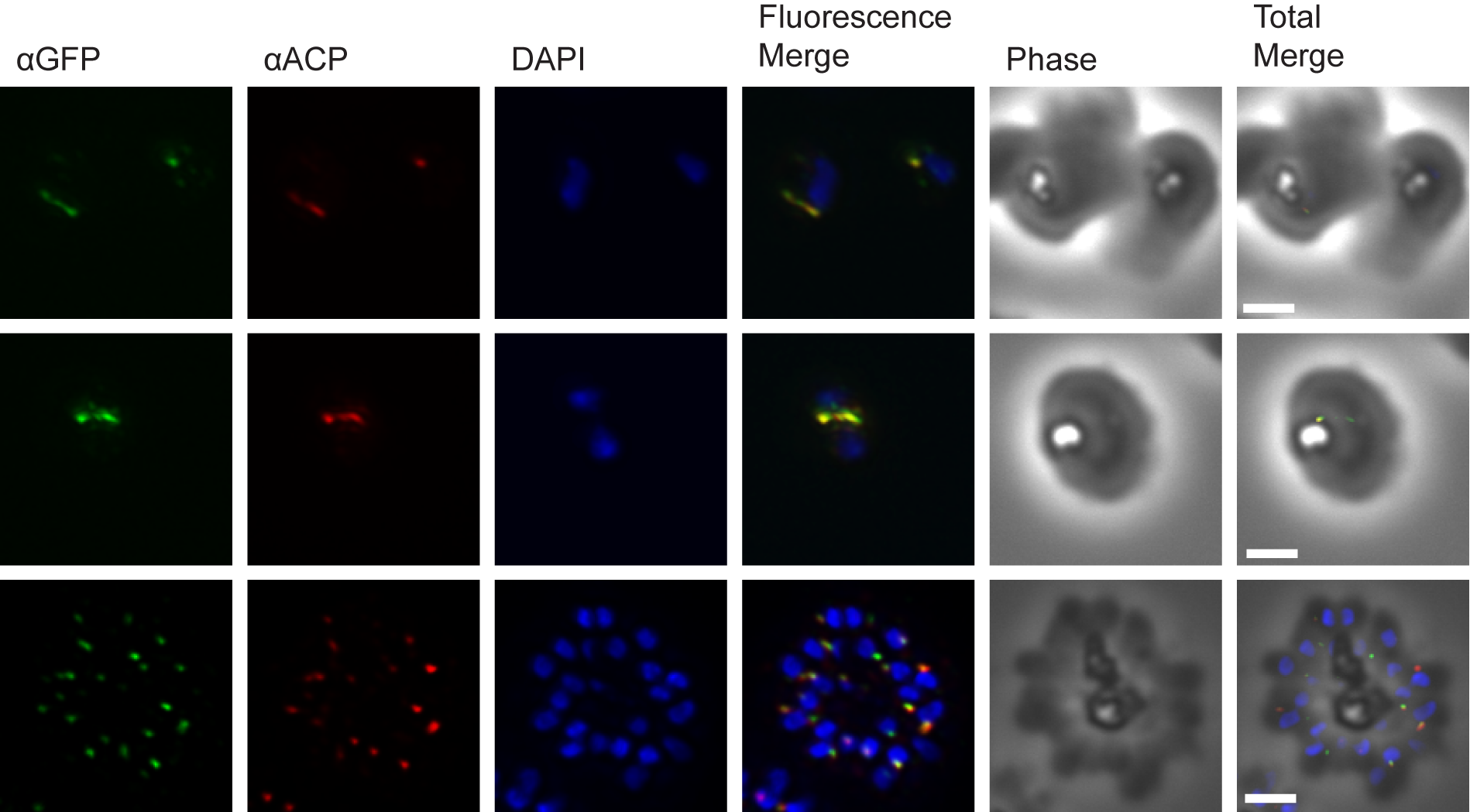

Supplement: Figure S2 — Immunofluorescence co-localization of SufEfl-GFP and endogenous ACP. An antibody specific for GFP co-localized with αACP antibodies, demonstrating apicoplast localization in late ring (top panel), late trophozoite or early schizont (middle), and schizont (bottom) stage parasites. The parasites were stained with DAPI to identify nuclei. Image z-stacks were deconvolved and then presented as a single combined image. Scale bar = 2 µm. (TIF) [file ppat.1003655.s002.tif]

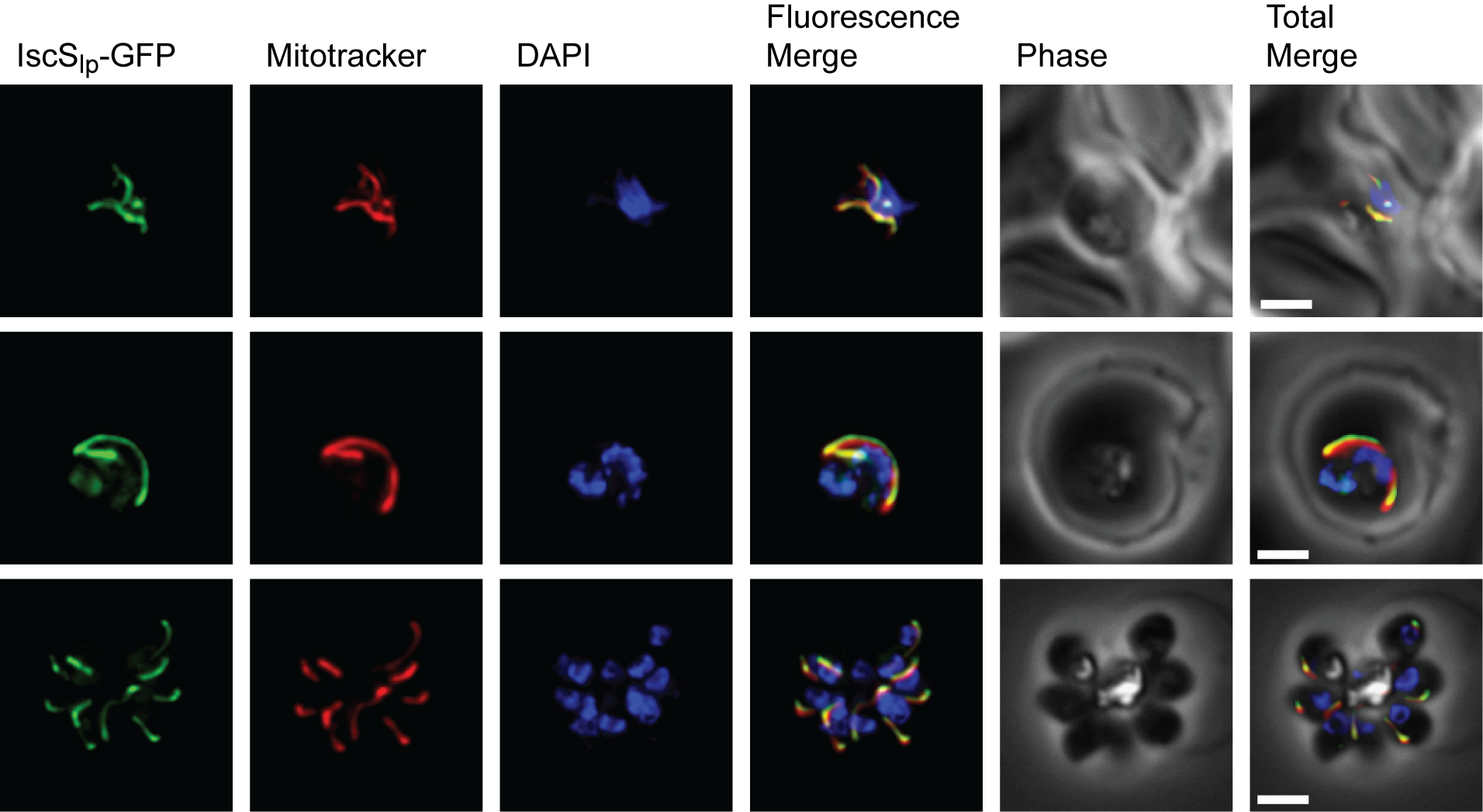

Supplement: Figure S3 — Subcellular localization of the IscS leader peptide to the mitochondrion of P. falciparum . Epifluorescent images of live P. falciparum erythrocytic-stage parasites expressing GFP fused to the leader peptide of IscS (amino acids 1–35). The parasites were stained with mitotracker to identify mitochondria and DAPI to identify nuclei. Image z-stacks were deconvolved and then presented as a single combined image. Scale bar = 2 µm. (TIF) [file ppat.1003655.s003.tif]

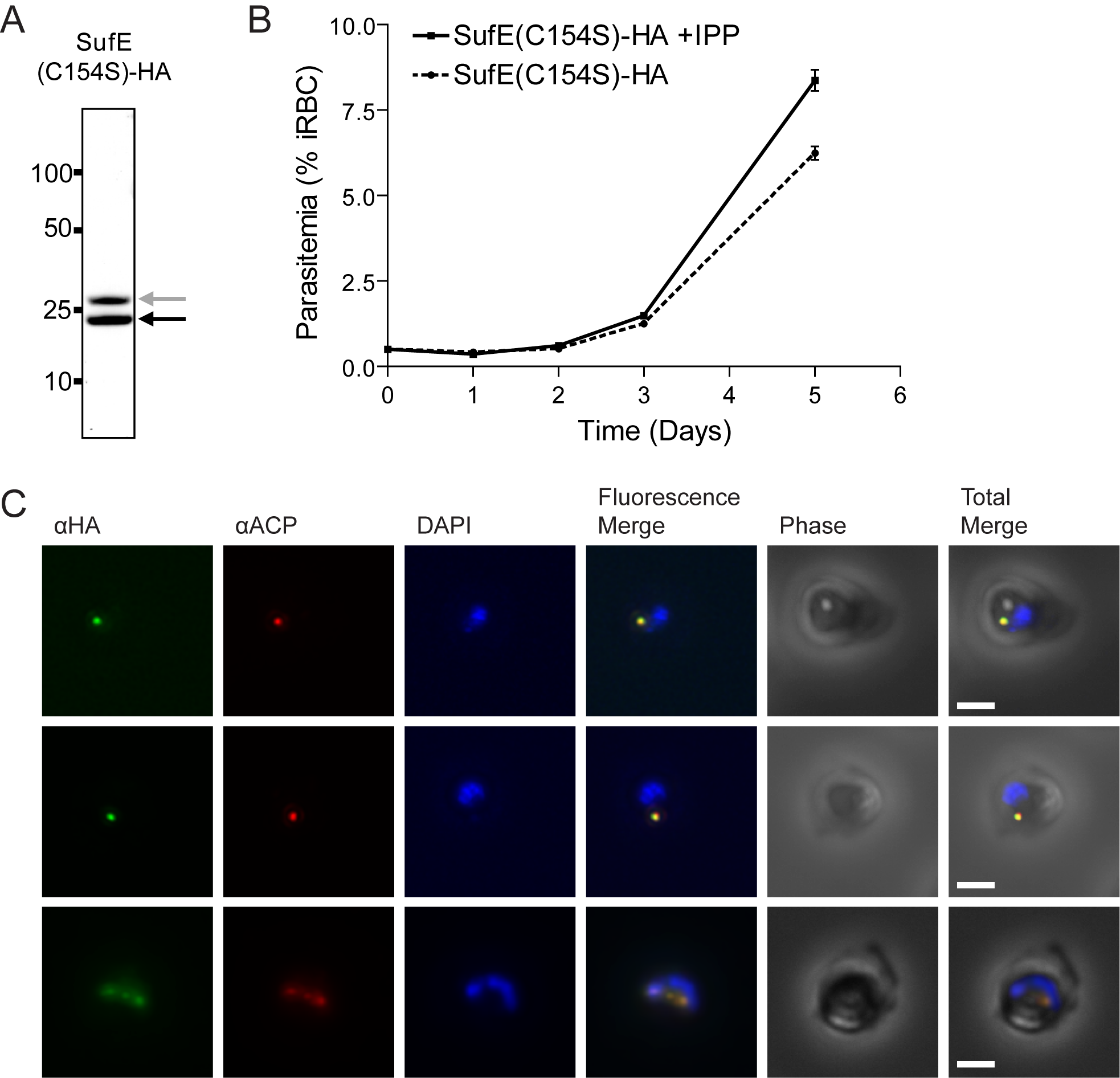

Supplement: Figure S4 — Overexpression of SufE(C154S)-HA in the apicoplast. A) Expression of SufE(C154S)-HA. An αHA western blot confirms the expression of the SufE(C154S)-HA construct and identifies the mature form (black arrow) as well as a minor population of unprocessed protein (grey arrow) prior to cleavage of the apicoplast transit peptide. B) IPP growth dependence of SufE(C154S)-HA parasites. SufE(C154S)-HA expressing parasites survive when supplemented with IPP (solid line) and when IPP is withdrawn (dashed line). Error bars represent SEM of triplicate measurements. C) Co-localization of SufE(C154S)-HA with endogenous ACP. Antibodies specific for the apicoplast marker ACP were used to visualize the apicoplast in blood stage parasites. Co-localization with an antibody specific for the HA tag shows that SufE(C154S)-HA is located in the apicoplast in late ring (top panel), trophozoite (middle), and early schizonts (bottom). Image z-stacks were deconvolved and then presented as a single combined image. Scale bar = 2 µm. (TIF) [file ppat.1003655.s004.tif]

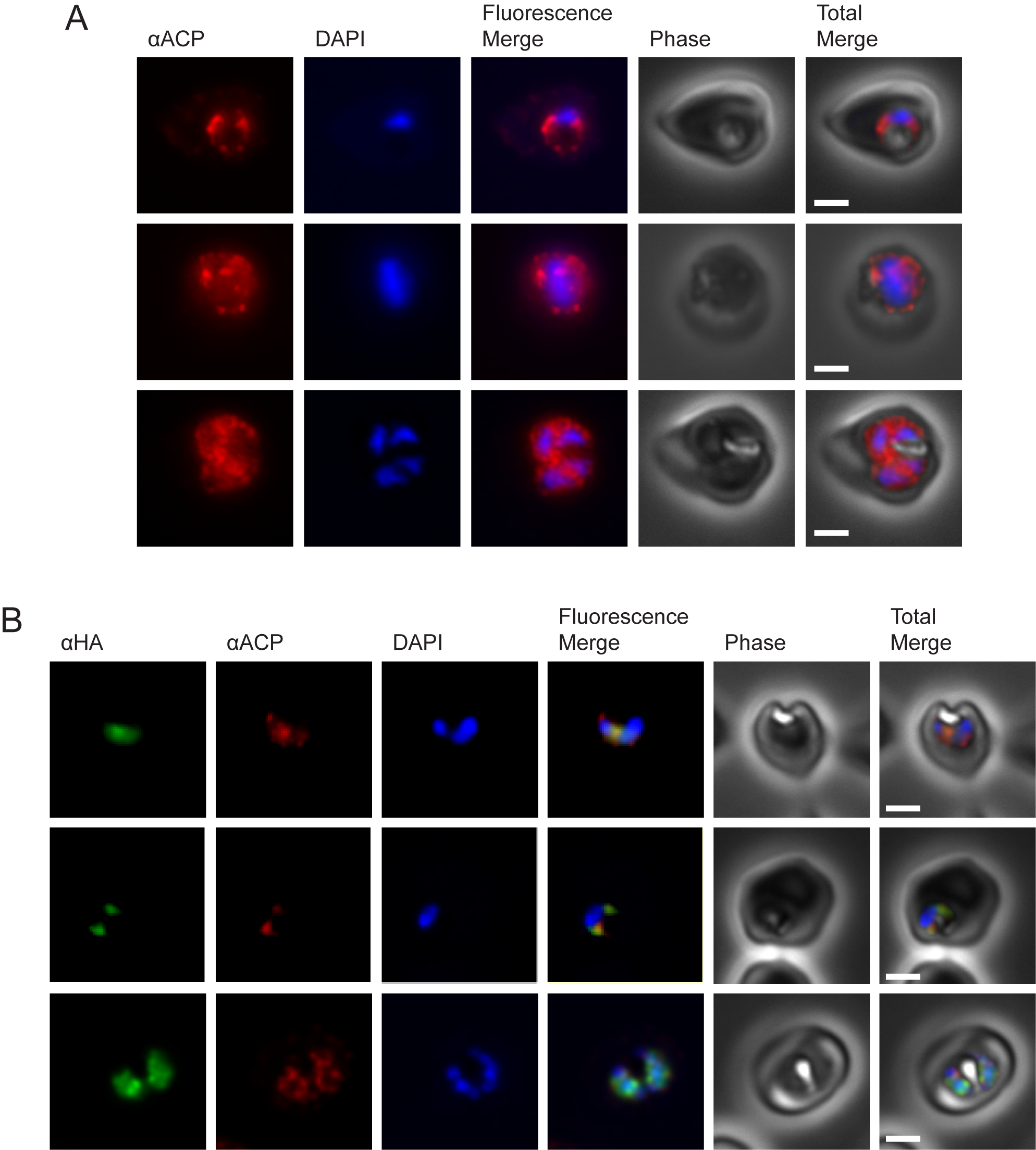

Supplement: Figure S5 — Subcellular localization of ACP and SufC(K140A)-HA shows disrupted apicoplast morphology. A) Localization of endogenous ACP in SufC(K140A)-HACaM parasites. Antibodies specific for the apicoplast marker ACP were used to visualize punctate vesicles in late ring (top panel), trophozoite or early schizont (middle), and schizont (bottom) stage parasites. B) Co-localization of SufC(K140A)-HA protein and endogenous ACP. An antibody specific for the HA tag indicates that SufC(K140A)-HA is co-localized with ACP in late ring (top panel), early schizont (middle), and schizont (bottom) stage parasites. Image z-stacks were deconvolved and then presented as a single combined image. Scale bar = 2 µm. (TIF) [file ppat.1003655.s005.tif]

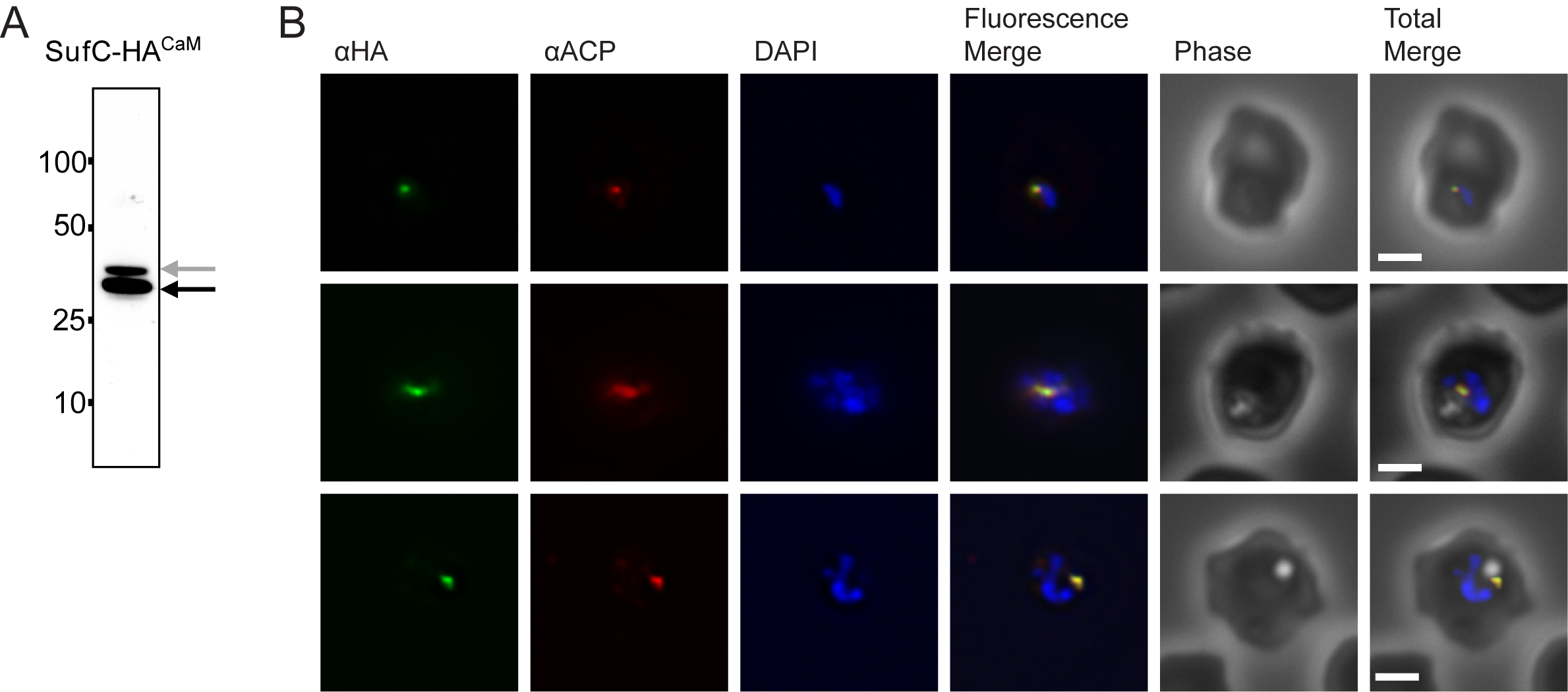

Supplement: Figure S6 — Subcellular localization of wildtype SufC to the apicoplast of P. falciparum . A) Expression of SufC-HACaM. An αHA western blot confirms the expression of the SufC-HA protein and identifies the mature form (black arrow) as well as a minor population of unprocessed protein (grey arrow) prior to cleavage of the apicoplast transit peptide. B) Co-localization of SufC-HA protein with endogenous ACP. An antibody specific for the HA tag co-localized with αACP antibodies, demonstrating apicoplast localization in late ring (top panel), late trophozoite or early schizont (middle), and schizont (bottom) stage parasites. The parasites were stained with DAPI to identify nuclei. Image z-stacks were deconvolved and then presented as a single combined image. Scale bar = 2 µm. (TIF) [file ppat.1003655.s006.tif]

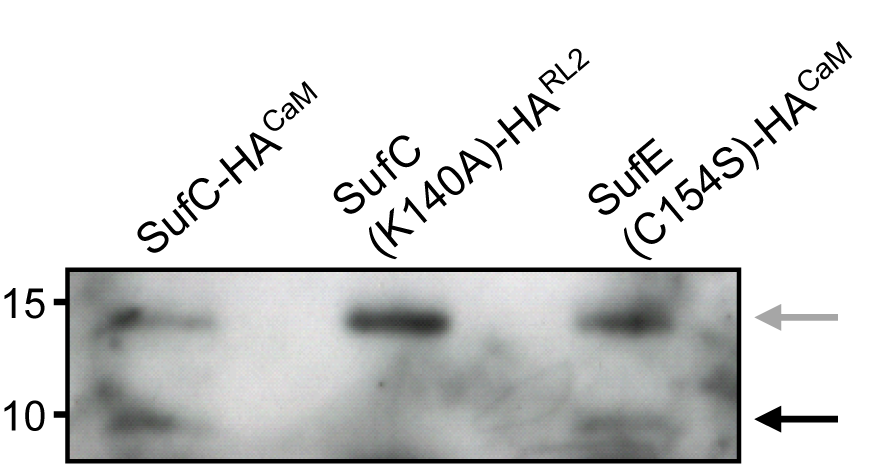

Supplement: Figure S7 — Processing of endogenous ACP. Antibodies specific for the Acyl Carrier Protein (ACP) were used to identify the mature form (black arrow) and the unprocessed protein (grey arrow) prior to cleavage of the apicoplast transit peptide. Only the unprocessed trafficking intermediate is present in the dominant negative SufC(K140A)-HARL2 parasite line, demonstrating loss of this apicoplast function. (TIF) [file ppat.1003655.s007.tif]

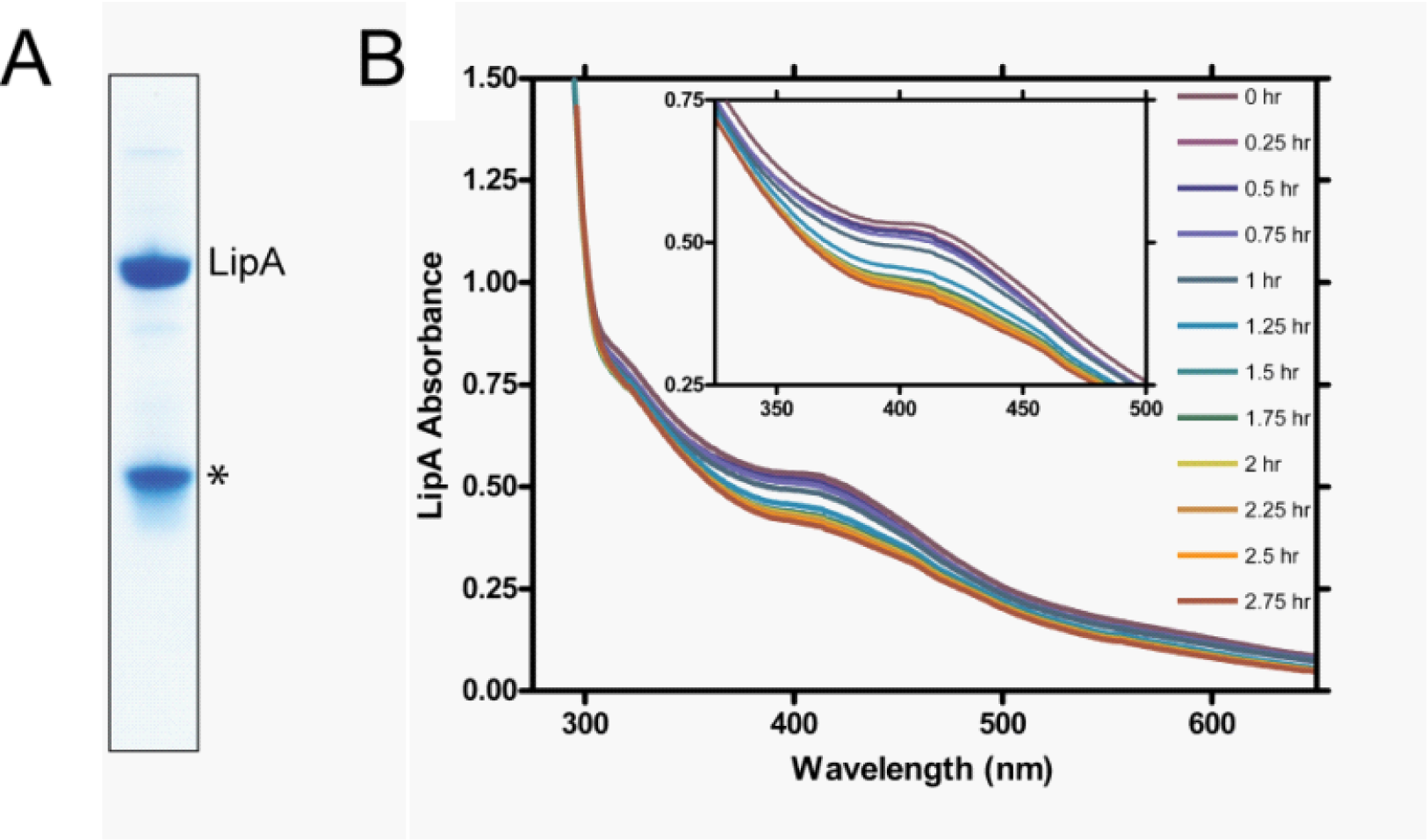

Supplement: Figure S8 — Purification and characterization of recombinant LipA protein. A) Lipoate synthase (LipA), was expressed as a GST fusion protein and purified from E. coli. Analysis by SDS-PAGE shows that GST-LipA89 migrates close to its predicted molecular weight of 63 kDa. Additional protein bands in the GST-LipA89 sample (asterisk) cross-react with antibodies specific for GST and likely result from proteolytic cleavage or incomplete translation. B) The UV-Vis absorption spectrum for anaerobically purified GST-LipA89 displays a broad peak at 440 nm typical of 4Fe-4S proteins. Because the LipA clusters are highly sensitive to oxygen, the signature 4Fe-4S UV-VIS signal degrades over time when exposed to air. (TIF) [file ppat.1003655.s008.tif]
