# Supplementary material for: The Suf Iron-Sulfur Cluster Synthesis Pathway Is Required for Apicoplast Maintenance in Malaria Parasites
Source: PLoS Pathog. 2013 Sep 26;9(9):e1003655. doi: 10.1371/journal.ppat.1003655 (PMC3784473; doi:10.1371/journal.ppat.1003655)
Supplement: Table S1 — DNA primers used in this study. The annealing portions of the sequences are underlined while the endonuclease sites are marked by boldface type. (DOC) [file ppat.1003655.s009.doc]

**Table S1.** DNA primers used in this study.

| **Gene** | **Primer Name** | **Sequence** |
| --- | --- | --- |
| IscS | IscS.AvrII.F | GGTGGT **CCTAGG** ATGAAATTTCTTCAAATAATAAAACATCTC |
|  | IscS.35.BsiWI.R | GGTGGT **CGTACG** GTTAATATTACTGATATGTTCATAGGTACGAC |
|  | IscS.fl.BsiWI.R | GGTGGT **CGTACG** TGTCCATATAAACTTTGGAATGTCGTC |
| Isd11 | Isd11.AvrII.F | GGTGGT **CCTAGG** ATGAATGGGAATCAAATAAAACAACTTAAG |
|  | Isd11.BsiWI.R | GGTGGT **CGTACG** TTTGTTAACTAAAGGTTTATCTACATG |
| SufS | SufS.TOPO.F | CACC ATGTTAAGAGGCCCTAGATGTC |
|  | SufS.TOPO.R | TCATTTTTCATTTTTCATTTCATTTAAC |
|  | SufS.ArvII.F | GGTGGT **CCTAGG** ATGTTAAGAGGCCCTAGATGTCTCTAC |
|  | SufS.59.BsiWI.R | GGTGGT **CGTACG** ATGATTATCTATATTAGGCTTTTCATCTTTTG |
|  | SufS.BamHI.F | GGTGGT **GGATCC** ATGATAGATTATTTTAAAAATGTAAGAGAAC |
|  | SufS.EcoRI.R | GGTGGT **GAATTC** TCATTTTTCATTTTTCATTTCATTTAAC |
| SufE | SufE.AvrII.F | GGTGGT **CCTAGG** ATGAATAAAAAGAAATTAAAAGCACATTTCTTTG |
|  | SufE.BsiWI.R | GGTGGT **CGTACG** ATTGTCCATATTCTTCAATATATTGGTGC |
| Insertion | RL2.F | GTGATATATTAGTAAGTTCATTTTACCAGTTAAGG |
|  | CaM.F | CTAGAAAAGGAATAACTAATATTTTATTTATTATCATTCAAG |
|  | GFP.R | CTTCACCCTCTCCACTGACAG |
|  | pLN.790.R | ACAAATGGTAATTCAAATAAAAGGTATAAATT |
| Integration | P1 | GATAAATGTATAAAAGATGAAACATGGTGAATC |
|  | P2 | GATAGCGATTTTTTTTACTGTCTG |
|  | P3 | GCACAGATGCGTAAGGAGAAAATACC |
|  | P4 | GCGCAATTAACCCTCACTAAAGGG |
| Cox1 | Cox1.Int.F | CTTCATCTTTAAGAATAATTGCACAAGAAAATGTAAATC |
|  | Cox1.Int.R | GGAAGCTTAGTATGGGTACATCATATGTAC |
| SufB | SufB.Int.F | GGTATAGAGGAGATTATTTAGGTAATGGTGG |
|  | SufB.Int.R | GACTCTGAAATACTTAAACCACGTTGC |
| SufS | SufS.Int.F | GTCATGCATCTAATGTTATTGG |
|  | SufS.Int.R | GTGCATCACTAGGTACAGG |
| mCherry | mCh.BsiWI.F | GGTGGT **CGTACG** AGCAAGGGCGAGGAGGATAAC |
|  | mCh.AflII.R | ACCACC **CTTAAG** TTACTTGTACAGCTCGTCCATGCC |
| LipA | LipA.EcoRI.F | GGTGGT **GAATTC** TACGCAAACAGATATGTACATG |
|  | LipA.PstI.R | GGTGGT **CTGCAG** TTAATTTTTTTTATCTTTGTTTCGTTG |
|  | LipA.BamHI.F | GGTGGT **GGATCC** TACGCAAACAGATATGTACATGAAGG |
|  | LipA.EcoRI.R | GGTGGT **GAATTC** TTAATTTTTTTTATCTTTGTTTCGTTGG |

The annealing portions of these sequences are underlined while the endonuclease sites are marked by boldface type.
